# Ligand conformational entropy as a regime-switching descriptor of the electrostatic–uptake relationship in nanocarrier–immune cell interactions

**DOI:** 10.1371/journal.pone.0358239

**Published:** 2026-09-18

**Authors:** Quynh Hoa Truong, Xuan Khanh Truong

**Affiliations:** Clevix Lab, Clevix LLC, Hanoi, Vietnam; Jinan University, CHINA

## Abstract

Surface charge is widely regarded as a primary determinant of nanoparticle–immune cell interactions, yet nanocarriers with similar physicochemical profiles often exhibit markedly different biological outcomes. Here, we show that ligand conformational diversity, quantified through an information-theoretic descriptor ($H_{ligand}$), is strongly associated with a regime transition in the relationship between surface charge and cellular uptake. Analysis of 105 gold nanoparticles from a benchmark protein corona dataset identifies a critical threshold at $H_{ligand}^{*}\approx 1.76$ bits (*F* = 47.3, *p* < 10^−14^): below this value, zeta potential is positively associated with uptake (*r* = +0.70), whereas above it the relationship reverses (r=−0.58), consistent with steric shielding by flexible surface ligands. Entropy-derived descriptors improve predictive performance across multiple model classes ($\Delta R^{2}$ up to +0.118), with gains concentrated in high-entropy regimes where physicochemical descriptors alone are insufficient. An analytical competition model captures this transition in a compact form (*R*^2^ = 0.550), with $2^{H^{*}}\approx 3.4$ effective microstates marking a numerical balance point between electrostatic and steric contributions. Cross-dataset validation on 652 multi-material nanoparticles supports transferability of the descriptor framework ($\Delta R^{2}=+0.181$). We emphasize that $H_{ligand}$ is a constructed information-theoretic descriptor rather than a thermodynamic entropy, and that all findings are derived from retrospective analysis of in vitro datasets and not from controlled experimental manipulation. The identified threshold therefore represents a reproducible statistical pattern and a testable hypothesis for how ligand flexibility modulates interaction regimes. This framework provides a computable basis for organizing nanocarrier design hypotheses and motivates prospective validation in more complex carrier systems.

## 1. Introduction

Nanomedicine has long promised precise control over therapeutic delivery by tuning nanoparticle size, surface chemistry, and targeting ligands. Yet, despite decades of optimization, immune recognition and clearance remain persistent obstacles to clinical translation [1]. Nanocarriers with nearly identical physicochemical specifications often exhibit markedly different biological fates, including divergent uptake, clearance kinetics, and immune activation profiles across tissues and individuals [2,3]. Such variability continues to limit reproducibility, scalability, and predictive design in nano-enabled therapeutics.

Current computational and experimental modeling strategies typically approach nano–immune interactions through static descriptors or correlation-driven learning, implicitly assuming stable mappings between nanoparticle properties and biological outcomes [4,5]. However, the nano–bio interface is intrinsically dynamic: protein coronas restructure, ligands reorient, cellular membranes fluctuate, and immune sensing pathways respond nonlinearly to transient cues [6,7]. As a result, models that neglect interaction-level uncertainty struggle to generalize across datasets and biological contexts, particularly when rare but biologically decisive immune events dominate outcomes.

In this work, we argue that nano–immune interactions can be productively described in terms of configurational uncertainty at the nano–bio interface, captured through information-theoretic descriptors derived from observable surrogates of microstate diversity. Rather than treating variability as noise, we frame it as an informative computable property of the interaction. Building on this perspective, we introduce an entropy-based, multi-scale modeling framework that explicitly represents interaction uncertainty through an Entropy-Oriented Interaction Metric (EOM). By integrating entropy-resolved descriptors across nanoparticle, cellular, and immune-response scales, the framework provides a principled basis for predictive, interpretable, and transferable modeling of nano–immune behavior, supporting rational design hypotheses for immune-safe nanocarriers.

## 2. Conceptual framework: configurational diversity at the nano–bio interface

Nano–immune interactions emerge from a complex coupling between nanoscale material properties, dynamic biological environments, and multilevel immune sensing mechanisms. At the nano–bio interface, nanoparticles do not encounter a static or uniform system, but rather a continuously evolving ensemble of microstates shaped by protein adsorption, molecular rearrangement, cellular mechanics, and immune surveillance [8,9]. These interactions unfold across multiple spatial and temporal scales, giving rise to variability that cannot be fully captured by fixed physicochemical descriptors alone.

A defining characteristic of this interface is configurational multiplicity. Protein coronas do not adopt fixed compositions but fluctuate across compartments; surface ligands fluctuate in orientation and accessibility; cellular membranes exhibit dynamic mechanical responses; and immune receptors integrate transient molecular cues over time. Each of these processes introduces uncertainty in how a given nanocarrier is perceived and processed by the immune system. Importantly, immune outcomes are often determined not by average interaction behavior, but by rare or transient configurations that cross activation thresholds, leading to clearance, tolerance, or targeting success [10,11].

Conventional nano–immune modeling frameworks have often treated such variability as experimental noise or sought to suppress it through averaging and correlation-based learning. While these approaches can achieve localized predictive success, they implicitly assume that interaction variability is extrinsic to the system. This assumption limits their capacity to generalize across datasets and biological contexts, particularly when stochastic interaction events dominate downstream immune responses.

From this perspective, interaction variability at the nano–bio interface is more appropriately described as a high-configurational-diversity phenomenon. Information-theoretic entropy provides a natural mathematical measure of configurational uncertainty, capturing the diversity of microstates accessible to a nanoparticle–biological system under given conditions [12]. In the context of nano–immune interactions, this descriptor reflects not disorder in a colloquial sense, but the breadth and dynamics of interaction configurations that influence immune recognition and response.

Framing nano–immune interactions through entropy descriptors shifts the modeling objective from identifying deterministic property–outcome mappings to characterizing interaction-level uncertainty. Immune outcomes can then be examined in relation to descriptor variation across scales, where specific regimes of configurational diversity may be associated with suppressed or amplified immune activation. This perspective aligns with emerging experimental observations in which subtle changes in corona composition, ligand presentation, or cellular context lead to disproportionate immune effects, despite minimal changes in nominal nanoparticle properties [13,14].

On this basis, we introduce an entropy-based conceptual framework in which nano–immune variability is captured by computable descriptors of interaction uncertainty alongside conventional static descriptors, rather than by static descriptors alone.

## 3. The Entropy-Oriented Interaction Metric (EOM)

The entropy-centered perspective outlined above motivates the need for a quantitative representation of interaction uncertainty that is both well-defined and biologically meaningful. To this end, we introduce the Entropy-Oriented Interaction Metric (EOM), a scale-consistent descriptor designed to capture configurational diversity at the nano–immune interface.

We emphasize at the outset that the quantities $H_{k}$ defined in this section are Shannon-type information measures computed over distributions of observable surrogates of microstate accessibility. They are constructed information-theoretic descriptors, not measurements of thermodynamic entropy or free energy. We retain the symbol *H* and the term *entropy* because the underlying mathematical objects are Shannon entropies; the interpretation, however, is descriptive rather than thermodynamic, and the clinical or biological meaning of any $H_{k}$ derives from what its surrogate distribution captures rather than from a direct thermodynamic readout.

Let 𝒮 denote the space of interaction microstates accessible under specified conditions. Formally, EOM is defined as an aggregate entropy measure over interaction-relevant microstate distributions:


$$\begin{array}{c} EOM=\sum_{k}w_{k}\;H_{k}\;, \end{array}$$ (1)

where $H_{k}$ represents entropy contributions associated with distinct interaction components, and $w_{k}$ are scale-dependent weighting factors.

### 3.1. Mathematical formulation of EOM components

**Protein-corona restructuring entropy (**$H_{corona}$**).** For a nanoparticle with *N* identified corona proteins, let $p_{i}$ denote the relative abundance of protein *i*, and let $\Delta p_{i}(t)$ represent its temporal fluctuation over observation window *T*. The corona restructuring entropy is computed as:


$$\begin{array}{c} H_{corona}=-\sum_{i=1}^{N}\left[{\bar{p}}_{i}\log_{2}{\bar{p}}_{i}+\alpha \cdot Var\left(\Delta p_{i}(t)\right)\right]\;, \end{array}$$ (2)

where ${\bar{p}}_{i}$ is the time-averaged abundance, and α is a scaling factor balancing compositional and temporal variability.

**Ligand presentation entropy (**$H_{ligand}$**).** Given a surface with *M* ligand sites, each with orientation vector ${𝐨}_{j}$ and accessibility $a_{j}$, the ligand presentation entropy incorporates spatial and orientational disorder:


$$\begin{array}{c} H_{ligand}=\frac{1}{M}\sum_{j=1}^{M}\left[-\sum_{\theta \in \Theta }P(\theta )\log_{2}P(\theta )\right]+\beta \cdot SD\{a_{j}\}\;, \end{array}$$ (3)

where P(θ) is the probability distribution of ligand orientations across discrete angular bins Θ, and β weights accessibility heterogeneity.

**Cellular mechanobiological coupling entropy (**$H_{mech}$**).** Cellular responses to nanoparticle engagement involve mechanical transitions across states $s\in {𝒮}_{mech}$. The entropy of these transitions is:


$$\begin{array}{c} H_{mech}=-\sum_{s\in {𝒮}_{mech}}\pi_{s}\log_{2}\pi_{s}+\gamma \cdot \left\langle \frac{\Delta E_{s}}{E_{0}}\right\rangle \;, \end{array}$$ (4)

where $\pi_{s}$ is the steady-state probability of mechanobiological state *s*, $\Delta E_{s}$ represents barrier-like quantities between states, *E*_0_ is a dataset-specific normalization constant rendering the second term dimensionless, and γ is a coupling coefficient. We emphasize that this is a constructed Shannon-type entropy supplemented by a dimensionless coupling term, and not a thermodynamic free-energy expression; for the datasets analyzed in this work, the second term is replaced by a curvature-based proxy combined with a Kullback–Leibler divergence over corona deviations (Section 6.2).

**Weight determination and normalization.** Weights $w_{k}$ are assigned based on scale-specific contributions to predictive performance, estimated via hierarchical regression on training data. The default weighting used in this work ($w_{corona}=0.5$, $w_{ligand}=0.25$, $w_{mech}=0.25$) reflects an in vitro context; the optimal weights are expected to depend on the biological scale of application, with corona-related contributions likely to carry greater weight at in vivo and tissue-level scales where biodistribution, opsonization, and clearance dominate immune outcomes. All entropy components are normalized to [0,1] using min-max scaling per dataset to ensure cross-dataset comparability.

Importantly, EOM features are applicable to a range of modeling architectures and data modalities. Within the datasets analyzed, low EOM values are typically associated with constrained interaction ensembles and reduced cellular engagement, whereas elevated EOM values are associated with broader microstate accessibility and more variable interaction outcomes [14,15]. The mechanistic causes underlying these statistical associations remain to be tested experimentally.

**General formulation versus practical estimators.** Equations 2–4 define the EOM components in their general form, in which the underlying probability distributions are taken over directly observable microstate quantities (e.g., orientation angles for $H_{ligand}$, mechanobiological state probabilities for $H_{mech}$, time-resolved corona compositions for $H_{corona}$). For the datasets analyzed in this work, several of these quantities are not directly available, and we therefore compute each component using observable surrogates that approximate the corresponding distributions: spectral-count Shannon entropy with a replicate-based temporal proxy for $H_{corona}$; a domain-informed ligand complexity score combined with |ζ| for $H_{ligand}$; and a curvature-based term combined with a Kullback–Leibler divergence over corona deviations for $H_{mech}$ (Section 6.2). The general equations should therefore be read as defining the formal targets of estimation, while the values used in all subsequent analyses are these dataset-specific estimators. Where estimators differ from the general form, the deviations and their justifications are stated explicitly in the corresponding Results subsections.

## 4. Materials and methods

### 4.1. Data sources and preprocessing

Two benchmark datasets were used. (i) The Walkey et al. (2014) supplementary dataset [16] was downloaded from the ACS Nano supporting information (nn406018q_si_001.xlsx), containing physicochemical properties, cell association, and LC-MS/MS spectral counts for 787 corona proteins across 105 gold NPs (after exclusion of 16 silver NPs from the original 121). (ii) The Ban et al. (2020) supplementary dataset [17] was downloaded from the PNAS supporting information, containing 652 data points across 40 + NP core materials and 178 proteins. Physicochemical features for the Walkey dataset comprised 8 descriptors: core size, $d_{H,synth}$, $d_{H,serum}$, $\Delta d_{H}$ (serum – synthesis), ${\text{PDI}}_{serum}$, $\zeta_{synth}$, $\zeta_{change}$ (serum – synthesis), and $\left|\zeta_{synth}\right|$. For the Ban dataset, 6 descriptors were available: core size, hydrodynamic diameter, zeta potential, |ζ|, $\Delta d_{H}$, and PDI.

**Scope of the cellular endpoints.** We note explicitly that the two datasets address complementary but non-identical aspects of nano–bio interactions. The Walkey dataset measures cell association in A549 human lung epithelial cells (a non-immune cell line) cultured in serum-containing medium, where the protein corona that mediates uptake includes immune-relevant opsonins (e.g., immunoglobulins, complement components, apolipoproteins). The Ban dataset reports the fractional composition of immune-related proteins within the protein corona across multiple nanoparticle types, providing a corona-level readout directly relevant to immune recognition. Throughout this work, we therefore use “nano–immune cell interaction” to refer to the broader nano–bio interface that governs immune-relevant outcomes, rather than to direct uptake measurements in immune cell lines. Interpretation of the findings should reflect this scope: the two datasets together capture immune-relevant signal at the corona and cell-association levels, but do not include direct uptake measurements in macrophages, dendritic cells, or other primary immune cells.

### 4.2. Computation of EOM

Entropy components were computed as described in Section 3: $H_{corona}$ (Shannon entropy of spectral count distribution), $H_{ligand}$ (information-theoretic encoding of ligand identity, zeta potential, and conformational complexity), and $H_{mech}$ (curvature-dependent mechanobiological entropy from hydrodynamic diameter distribution). All components were *z*-score normalized prior to modeling.

### 4.3. Interpretation of $H_{ligand}$

We emphasize that $H_{ligand}$ is an information-theoretic *proxy* for ligand conformational diversity, not a direct thermodynamic measurement of conformational entropy ($\Delta S_{conf}$). The heuristic scoring system encodes domain knowledge about ligand flexibility, molecular weight, and charge character into a scalar complexity measure. Post hoc validation using RDKit molecular descriptors (Supporting Information) confirms that this proxy primarily captures the number of rotatable bonds (*r* = 0.85 with original scoring), which is a well-established correlate of conformational freedom. The predictive improvement is robust to alternative scoring schemes (Table 1), supporting the interpretation that $H_{ligand}$ captures genuine variation in ligand conformational properties rather than scoring artifacts.

**Table 1:** Sensitivity of prediction performance (*R*^2^, LOO-CV) to $H_{ligand}$ scoring scheme. All schemes use Random Forest with physicochemical + EOM features. This sensitivity analysis is reported using the earlier LOO-CV protocol as a secondary check; the primary model comparisons reported elsewhere in this manuscript use 10-fold cross-validation repeated over 20 random seeds.

Scheme	Description	*R* ^2^	Pearson *r*
Original	PEG = 3.5, citrate = 0.8, CTAB = 1.8	0.603	0.777
Uniform	All ligands = 2.0	0.535	0.732
Binary	PEG = 3.0, all others = 1.0	0.590	0.768
Reversed	PEG = 0.8, citrate = 3.5	0.588	0.766
Expanded	PEG = 5.0, citrate = 0.5	0.602	0.776
Phys. only baseline (no EOM)		0.542	0.730

### 4.4. Modeling and cross-validation

Four regression algorithms were evaluated: Ridge regression (α optimized by nested CV), support vector regression (SVR, RBF kernel, *C* and ϵ optimized), *k*-nearest neighbors (kNN, *k* optimized), and Random Forest (RF, 500 estimators) [18]. The primary evaluation protocol was 10-fold CV repeated over 20 random seeds, with global out-of-fold (OOF) *R*^2^ computed by concatenating all held-out predictions and evaluating once per seed. Feature standardization (zero mean, unit variance) was applied within each fold to prevent information leakage. Statistical significance was assessed by paired *t*-tests across the 20 seeds (Phys. vs. Phys. + EOM), with 95% bootstrap confidence intervals repor*t*ed.

Note on feature count: When the EOM composite score is included as a feature alongside the three individual entropy components, the total is 12 features (8 physicochemical + 4 entropy). For Ridge, SVR, and kNN, the EOM composite was excluded due to its collinearity with the individual components, yielding 11 features. RF, which is robust to collinear inputs, used all 12.

### 4.5. Advanced analyses

The critical entropy threshold (Section 6.8) was identified by grid-search piecewise linear regression of $\log_{2}$ cell association on $\zeta_{synth}$, with $H_{ligand}$ as the splitting variable; significance assessed by *F*-test against a single-slope null model, with bootstrap resampling (2,000 iterations) for threshold confidence intervals. Regime-stratified performance (Section 6.10) used tercile splits on $H_{ligand}$ with 5-fold CV × 3 seeds within each stratum. Shapley variance decomposition (Section 6.11) evaluated all 3! = 6 orderings of entropy component addition over 5 seeds, computing mean marginal $\Delta R^{2}$ as the Shapley value for each component [19].

### 4.6. Reproducibility

All computational procedures were implemented in Python 3.10 using scikit-learn 1.3, NumPy, SciPy, and pandas. The complete analysis pipeline, including data preprocessing scripts, entropy computation modules, model training routines, cross-validation protocols, and figure generation code, is openly available at https://www.github.com/ClevixLab/Nano. The repository includes step-by-step instructions to reproduce all numerical results, tables, and figures reported in this manuscript.

## 5. Multi-scale modeling architecture

**Fig 1:**
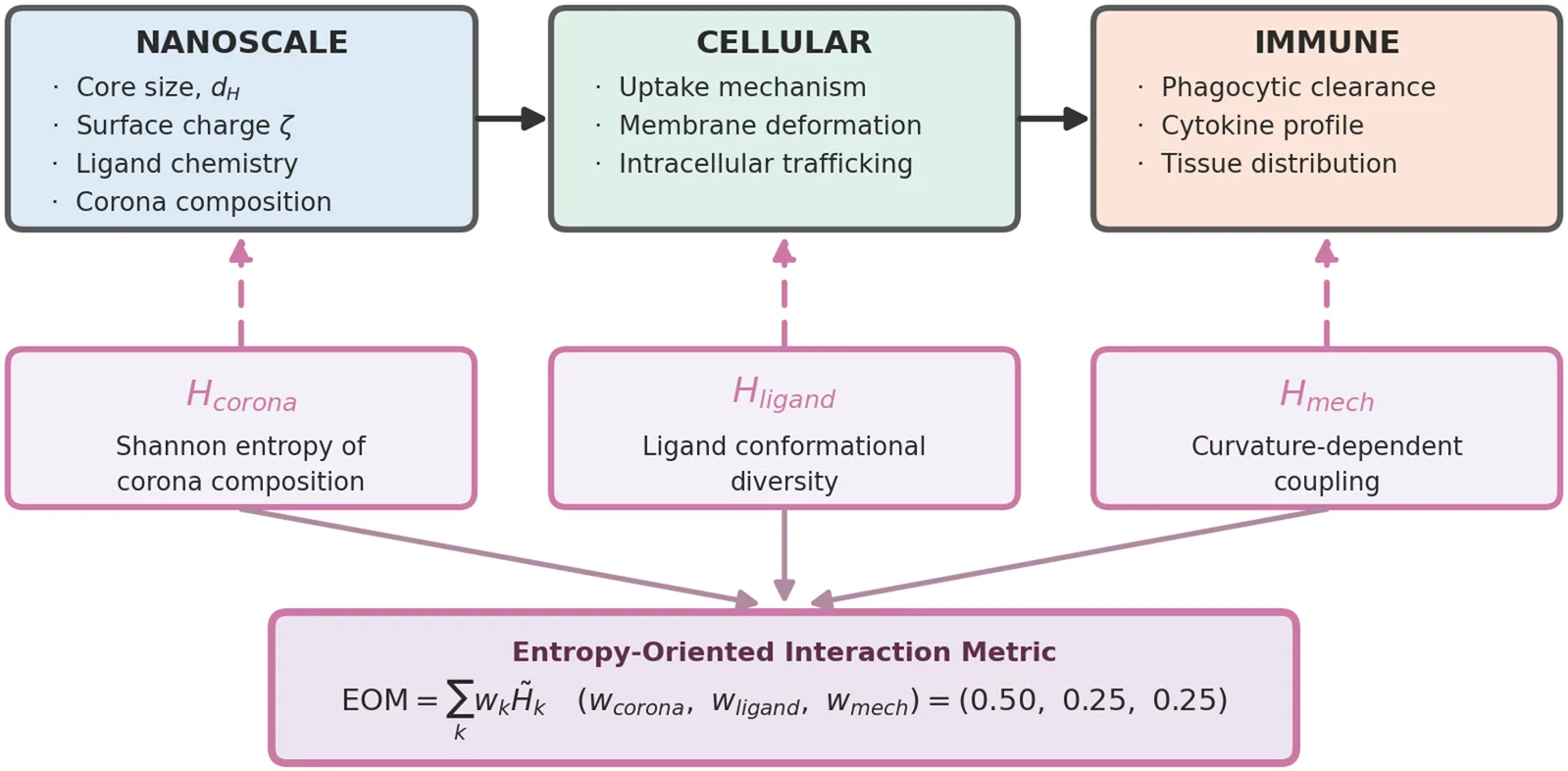
Multi-scale modelling architecture for entropy-augmented nano–immune interaction prediction. Three coupled levels (nanoscale, cellular, immune) represent hierarchically organised interaction properties, linked by the biological cascade from nanoparticle properties through cellular uptake to immune-level outcomes (solid arrows). Three information-theoretic descriptors are attached to the scale at which each is defined (dashed arrows): $H_{corona}$ (Shannon entropy of corona composition), $H_{ligand}$ (ligand conformational diversity) and $H_{mech}$ (curvature-dependent coupling). The descriptors are *z*-score standardised and aggregated into the composite EOM score (Eq 1) using the default in vitro weighting $\left(w_{corona},w_{ligand},w_{mech})=(0.50,0.25,0.25\right)$.

The entropy-oriented framework requires a modeling architecture capable of integrating heterogeneous information across scales while preserving interaction-level meaning. We organize the modeling framework into three coupled but conceptually distinct levels (Fig 1):

1. **Nanoscale Level:** Represents particle-specific properties including size, charge, hydrophobicity, and EOM components related to corona restructuring and ligand presentation.
2. **Cellular Level:** Encodes uptake mechanisms, membrane deformation energy, and intracellular trafficking patterns, incorporating mechanobiological entropy.
3. **Immune Level:** Captures system-level responses including phagocytic clearance rates, cytokine profiles, and tissue distribution.

### 5.1. Model implementation

A central design principle of the EOM framework is model agnosticism: the entropy-derived features encode interaction-level information in a compact, interpretable form and can augment any supervised learning pipeline. We compare four algorithms spanning distinct inductive biases:

- **Ridge Regression**—a linear baseline testing whether EOM features contribute additive predictive signal.
- **Support Vector Regression (SVR)**—a kernel-based method capable of capturing nonlinear relationships through implicit feature mapping.
- ***k*-Nearest Neighbors (kNN)**—a purely distance-based baseline that tests whether entropy features improve local similarity structure in feature space.
- **Random Forest (RF, 500 estimators)**—a parallel ensemble method with built-in feature importance estimates, robust to collinear inputs.

This model-agnostic strategy serves two purposes: (i) it demonstrates that predictive improvements arise from the entropy representation itself rather than from a particular model class, and (ii) it reveals model-dependent sensitivity to entropy features, providing interpretive insight into when entropy-derived features improve prediction.

### 5.2. Training and validation strategy

- **Cross-Validation:** 10-fold cross-validation with 20 independent random splits constitutes the primary evaluation protocol. For each run, out-of-fold predictions are concatenated across all folds and a single global *R*^2^ is computed, avoiding the downward bias of averaging per-fold *R*^2^ values [18]. Results are reported as mean ± standard deviation across 20 runs. Leave-one-out cross-validation (LOO-CV) results are provided in the Supporting Information as a sensitivity check.
- **Feature Sets:** Three primary feature configurations are compared: physicochemical only (8 features: core size, $d_{H,synth}$, $d_{H,serum}$, $\Delta d_{H}$, ${\text{PDI}}_{serum}$, $\zeta_{synth}$, Δζ, |ζ|), EOM entropy features only (4 features: $H_{corona}$, $H_{ligand}$, $H_{mech}$, EOM composite), and physicochemical + EOM (11 or 12 features; see Materials and Methods for details on feature count by model).
- **Metrics:** *R*^2^ (coefficient of determination), Mean Absolute Error (MAE), and Root Mean Squared Error (RMSE).
- **Statistical Testing:** Paired *t*-tests on seed-level global *R*^2^ values assess whether EOM-augmented models yield statistically significant improvement. Bootstrap 95% confidence intervals (10,000 resamples) for $\Delta R^{2}$ are reported.
- **Preprocessing:** All continuous features are standardized (*z*-score) within each cross-validation fold to prevent information leakage.

## 6. Results

### 6.1. Dataset description

The dataset comprises 105 distinct gold nanoparticles (NPs) characterized by three core sizes (15, 30, and 60 nm), 10 surface ligands spanning anionic (*n* = 57), cationic (*n* = 27), and neutral (*n* = 21) charge classes, and complete physicochemical profiles including hydrodynamic diameter ($d_{H}$) and zeta potential (ζ). Protein corona composition was measured by LC-MS/MS, yielding spectral counts for 787 unique proteins across all 105 NPs. Cell association was quantified via flow cytometry (A549 human lung epithelial cells), following the original experimental protocol described in [16].

### 6.2. Computation of EOM on experimental data

Entropy components were computed directly from experimental measurements as follows:

**Corona formation entropy ($H_{corona}$).** Shannon entropy was computed over the normalized protein abundance vector (RPA%) for each nanoparticle according to Eq. 2. Since temporal data were not available, the temporal variability term was approximated using the coefficient of variation across NP replicates sharing the same core–ligand identity. Values ranged from 2.01 to 3.85 bits (mean 3.35±0.30).

**Ligand presentation entropy (**$H_{ligand}$**).** In the absence of atomistic ligand orientation data, $H_{ligand}$ was estimated using a two-component proxy: (i) a ligand-specific surface chemistry complexity score, and (ii) the absolute zeta potential |ζ| as an indicator of charge-driven surface heterogeneity. Values ranged from 1.05 to 3.71 (mean 2.03±0.68).

**Mechanobiological coupling entropy (**$H_{mech}$**).** $H_{mech}$ was computed from curvature-dependent entropy (inversely related to $d_{H}$) and a Kullback–Leibler divergence term quantifying each NP’s corona deviation from the population mean corona profile. Values ranged from 0.29 to 0.69 (mean 0.50±0.09).

**EOM composite score.** The composite EOM score was computed as a weighted combination ($w_{corona}=0.5$, $w_{ligand}=0.25$, $w_{mech}=0.25$), with all components *z*-score normalized prior to aggregation.

### 6.3. Entropy–outcome correlations

We first examined bivariate correlations between each entropy component and $\log_{2}$-transformed cell association (Table 2).

**Table 2:** Pearson correlations between EOM entropy components and $\log_{2}$ cell association (*n* = 105 gold NPs). Significant correlations at *p* < 0.001 are indicated in bold.

Entropy Component	Pearson *r*	*p*-value	Interpretation
$H_{corona}$	+0.275	$4.5\times 10^{-3}$	Weak positive
$H_{ligand}$	−0.578	$1.1\times 10^{-10}$	Strong negative
$H_{mech}$	−0.396	$2.9\times 10^{-5}$	Moderate negative
EOM (composite)	−0.189	$5.3\times 10^{-2}$	Weak negative

**Fig 2:**
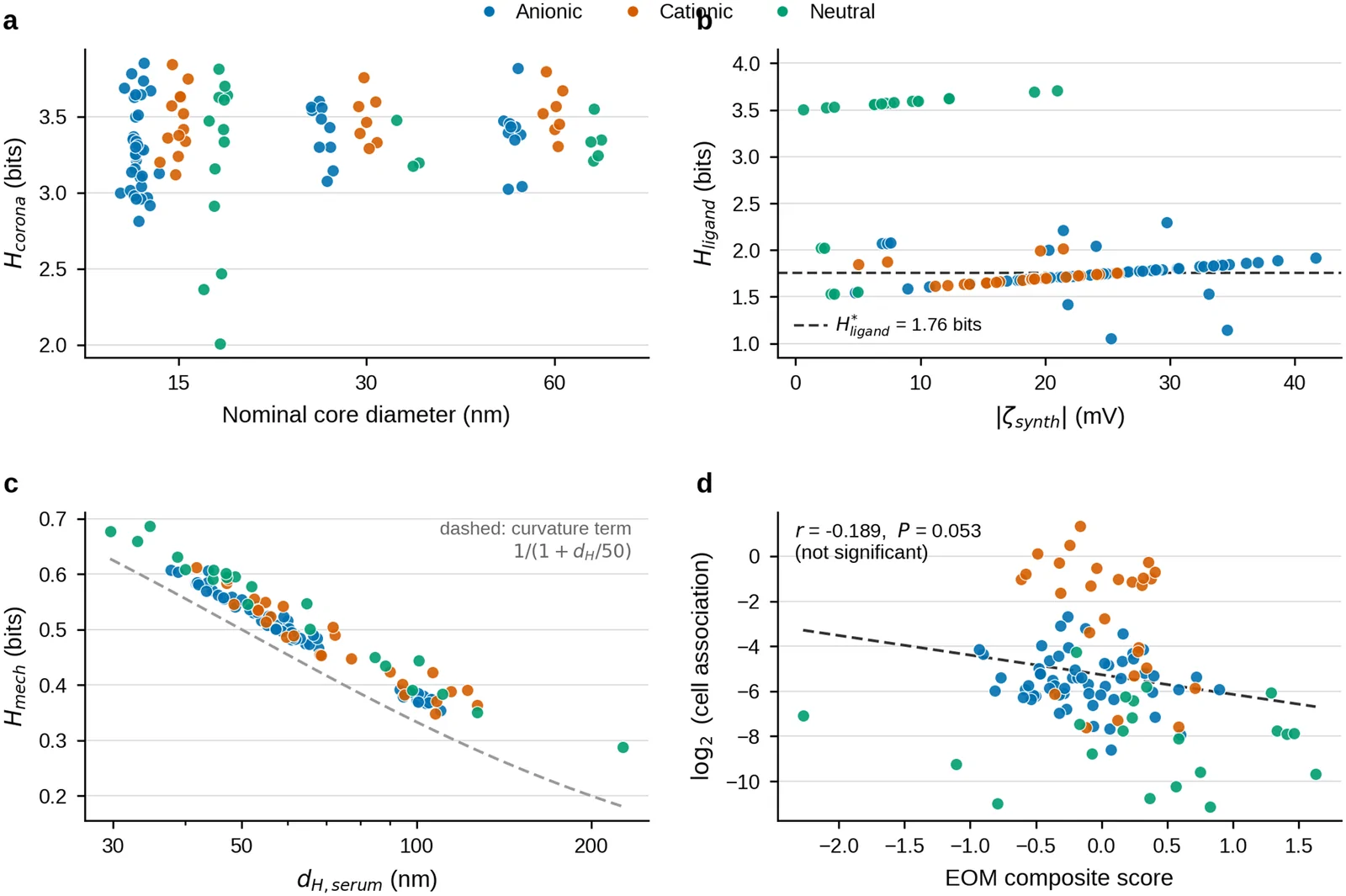
Distribution of EOM entropy descriptors across the 105 gold nanoparticles, coloured by surface charge class. **(a)** $H_{corona}$ by nominal core diameter; 15 nm particles span the widest range, while 30 and 60 nm particles converge near the upper end. **(b)** $H_{ligand}$ versus $\left|\zeta_{synth}\right|$; neutral, PEG-dominated particles occupy the highest values. The dashed line marks the regime boundary $H_{ligand}^{*}=1.76$ bits identified in Section 6.8. **(c)** $H_{mech}$ versus hydrodynamic diameter (logarithmic axis); the dashed curve is the curvature term $1/(1+d_{H}/50)$ that dominates the descriptor. **(d)** Composite EOM score versus $\log_{2}$ cell association; the dashed line is the least-squares fit (r=−0.189, *P* = 0.053), a weak negative trend that does not reach significance.

The strongest individual predictor was ligand presentation entropy ($H_{ligand}$: r=−0.578, $p=1.1\times 10^{-10}$), indicating that nanoparticles with higher surface chemistry complexity tend to exhibit lower cell association. This negative correlation is consistent with the well-established PEG stealth effect [2]. The distribution of entropy components across charge classes and core sizes is shown in Fig 2.

### 6.4. Predictive model comparison

**Fig 3:**
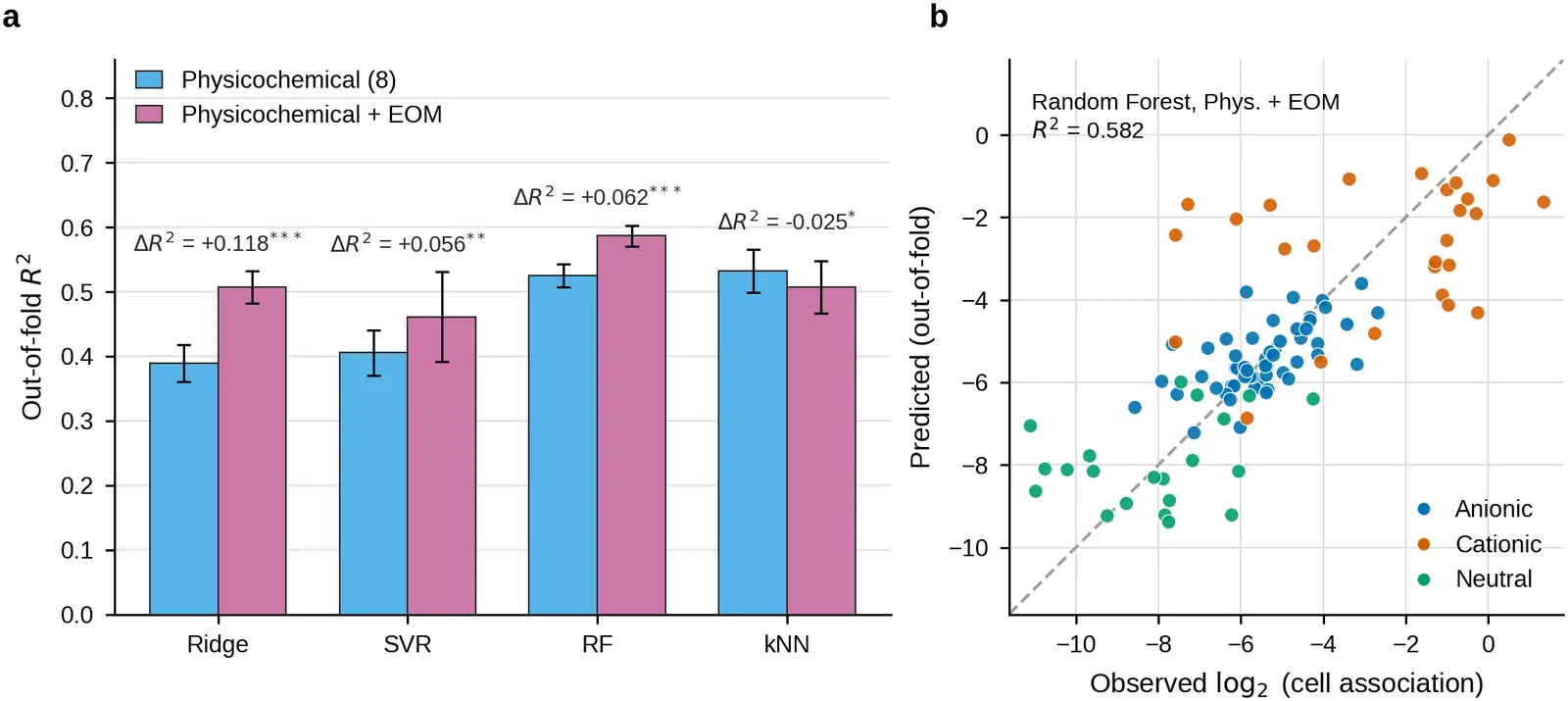
Predictive performance for $\log_{2}$ cell association (*n* = 105). **(a)** Global out-of-fold *R*^2^ for physicochemical features alone versus physicochemical + EOM across four model classes. Bars show the mean over 10-fold cross-validation repeated across 20 random seeds; error bars show ±1 SD across seeds. $\Delta R^{2}$ is annotated above each pair; asterisks denote paired *t*-test significance across seeds (^***^P<0.001, ^**^P<0.01, ^*^P<0.05). Ridge, SVR and Random Forest improve, whereas kNN degrades. **(b)** Out-of-fold predicted versus observed values for the best-performing model (Random Forest, physicochemical + EOM) for a single representative seed (*R*^2^ = 0.582; the 20-seed mean is 0.587±0.016). Poin*t*s are coloured by surface charge class and the dashed line is the 1:1 identity.

To quantify the added predictive value of EOM-derived features, we evaluated three feature sets using four regression algorithms under 10-fold cross-validation with 20 independent random splits (*n* = 105). Results are summarized in Table 3 and Fig 3.

**Table 3:** Prediction performance for $\log_{2}$ cell association (10-fold CV, 20 runs, global OOF *R*^2^, *n* = 105). $\Delta R^{2}$: improvement from Phys. only to Phys. + EOM. Statistical significance assessed by paired *t*-test across 20 runs; 95% CI from bootstrap (10,000 resamples). Feature counts: Ridge, SVR, and kNN use 11 features (8 Phys. + 3 EOM components); RF uses 12 features (8 Phys. + 3 EOM + EOM composite). See Materials and Methods for rationale.

Model	Phys. only	Phys. + EOM	$\Delta R^{2}$	95% CI	p
Ridge	0.389±0.029	0.507±0.025	+0.118	[0.107, 0.130]	$8.0\times 10^{-14}$
SVR	0.405±0.036	0.461±0.069	+0.056	[0.025, 0.083]	$1.7\times 10^{-3}$
RF	0.525±0.018	0.587±0.016	+0.062	[0.056, 0.068]	$3.4\times 10^{-14}$
kNN	0.532±0.033	0.508±0.041	−0.025	[−0.042,−0.007]	$1.7\times 10^{-2}$

**Key findings.**

(i) **EOM yields stable predictive improvements across three of four model classes.** Ridge regression exhibits the largest absolute gain ($\Delta R^{2}=+0.118$, 95% CI: [0.107, 0.130]), consistent with entropy features providing linear separability that the physicochemical baseline lacks. Random Forest achieves the best absolute performance ($R^{2}=0.587\pm 0.016$) with a stable gain of $\Delta R^{2}=+0.062$ (CI: [0.056, 0.068]). SVR shows a moderate but consistent improvement ($\Delta R^{2}=+0.056$, CI: [0.025, 0.083]). Ridge and RF yield *p* < 10^-13^, and SVR *p* < 0.01, by paired *t*-test across 20 runs. Because repeated CV splits on a single dataset are not fully independent statistical units, these *p*-values are interpreted as evidence of stability across resampling ra*t*her than as independent biological replication.
(ii) **kNN exhibits significant degradation.** Adding entropy features to kNN reduces performance by $\Delta R^{2}=-0.025$ ($p=1.7\times 10^{-2}$). This is attributable to two factors: (i) the curse of dimensionality—expanding from 8 to 11 features dilutes local neighborhood structure in a sample of only *n* = 105; and (ii) scale heterogeneity—entropy features operate on different distributional scales than physicochemical descriptors (e.g., $H_{ligand}$ is bimodally distributed with a PEG/non-PEG gap), distorting Euclidean distances that kNN relies upon. This finding provides indirect evidence that entropy signal operates through structured feature interactions (captured by Ridge, SVR, and RF) rather than through simple proximity in feature space.
(iii) **Model-class independence rules out overfitting.** Significant improvement across linear (Ridge), kernel (SVR), and ensemble (RF) architectures indicates that the predictive gain arises from the entropy representation itself, not from algorithmic artifacts.
(iv) **Raw corona proteins fail as standalone predictors.** The top-20 most variable corona proteins yield near-zero predictive performance (RF, *R*^2^ = 0.095; see Supporting Information Table S2 in S1 File), indicating that raw spectral counts lack the structural information needed to predict cell association without entropy-derived contextualization.

### 6.5. Sensitivity analysis for ligand complexity scores

The *R*^2^ range across all five schemes (0.535–0.603) consistently exceeds or matches the physicochemical-only baseline (0.542), demonstrating that the predictive benefit of EOM is robust to the specific numerical parameterization of $H_{ligand}$.

### 6.6. Design-space analysis

**Fig 4:**
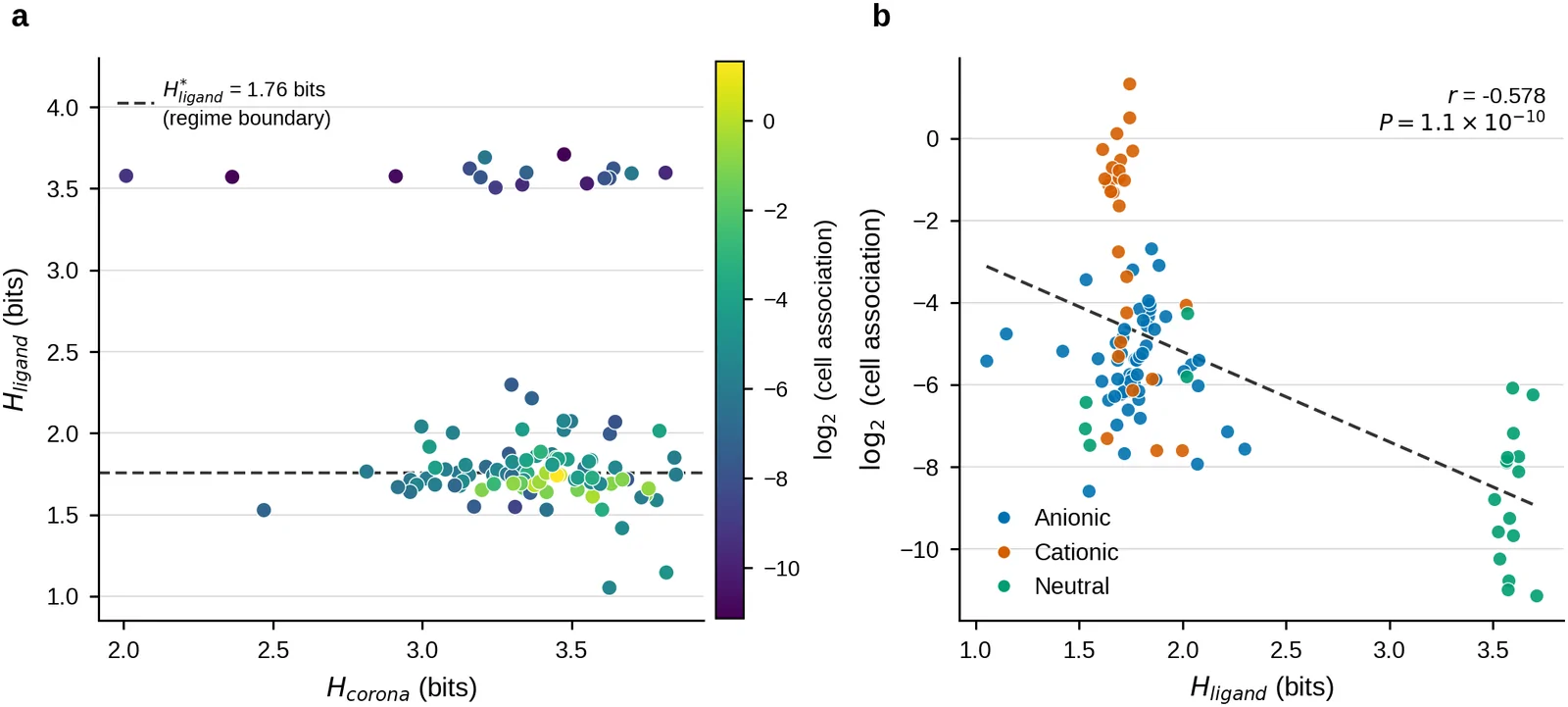
Entropy-resolved design space for the 105 gold nanoparticles. **(a)** $H_{ligand}$ versus $H_{corona}$ with points coloured by $\log_{2}$ cell association; the dashed line marks $H_{ligand}^{*}=1.76$ bits, separating the low- and high-$H_{ligand}$ statistical regimes, interpreted respectively as more electrostatically responsive and more sterically shielded. **(b)** $H_{ligand}$ versus $\log_{2}$ cell association, the strongest bivariate association in the dataset (r=−0.578, $P=1.1\times 10^{-10}$). Neutral (PEG-dominated) particles combine the highest $H_{ligand}$ with the lowest cell association; cationic particles show the opposite pattern.

Projecting all 105 NPs into the entropy space reveals structured interaction regimes (Fig 4). NPs with high ligand entropy and low corona entropy tend to exhibit low cell association, consistent with PEG-driven stealth behavior. NPs with low $H_{ligand}$ span a wide range of uptake values, reflecting the influence of other interaction factors.

### 6.7. Entropy landscape by NP properties

**Fig 5:**
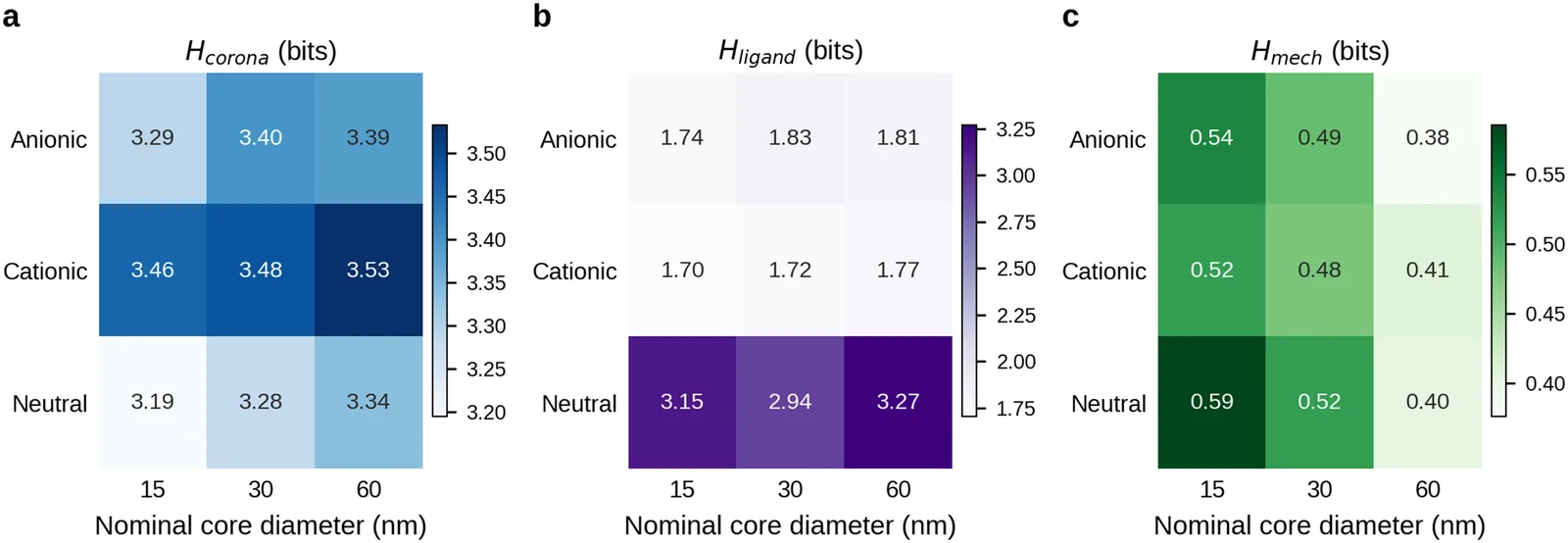
Entropy landscape by surface charge class and nominal core diameter. Each panel shows the mean value of one descriptor across the nine charge–size combinations, annotated in every cell. **(a)** $H_{corona}$. **(b)** $H_{ligand}$: neutral particles are highest at every core size, consistent with PEG conformational flexibility, and anionic particles are lowest. **(c)** $H_{mech}$: the smallest cores are highest within every charge class, consistent with the curvature dependence of the descriptor. Cells marked n/a contain no particles of that combination.

The entropy heatmap (Fig 5) reveals systematic patterns in entropy components across charge classes.

### 6.8. Critical entropy threshold: regime reversal in the electrostatic–uptake relationship

The preceding analyses demonstrate that entropy features improve prediction on average. A deeper question is whether the entropy contribution is uniform across the dataset or concentrated in specific interaction regimes. We address this through three complementary analyses.

**Fig 6:**
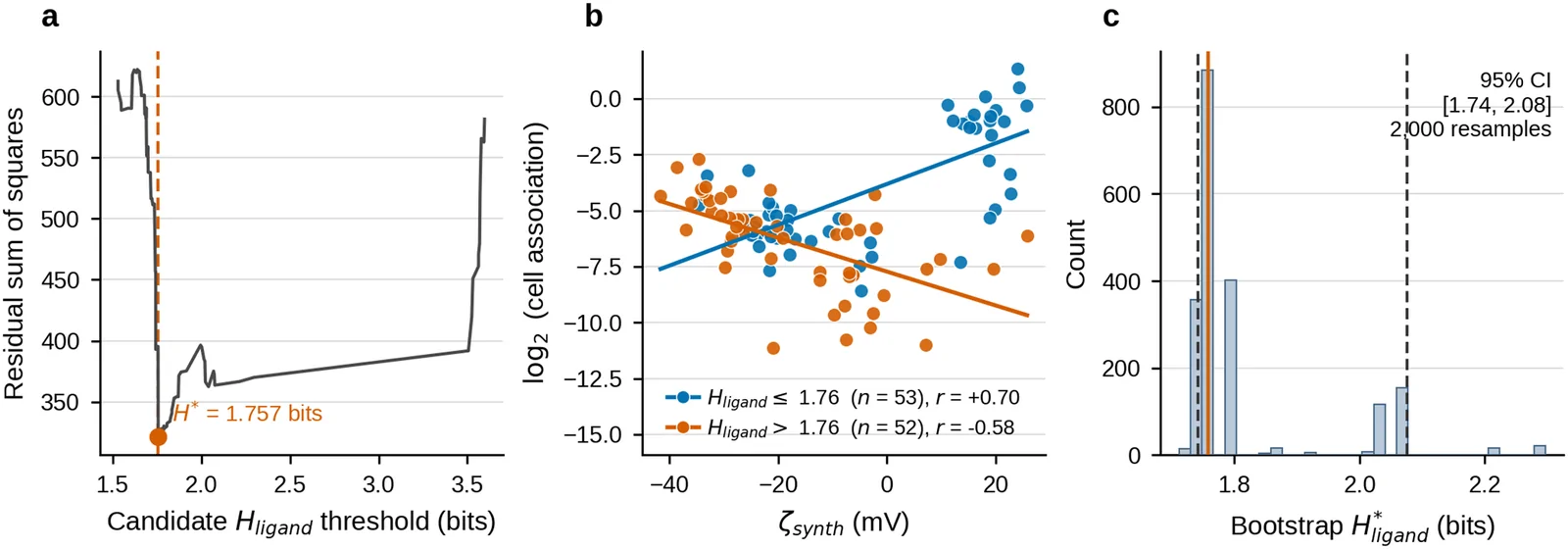
Critical entropy threshold in the electrostatic–uptake relationship. **(a)** Breakpoint search: residual sum of squares of the piecewise model as a function of the candidate $H_{ligand}$ threshold, minimised at $H_{ligand}^{*}=1.757$ bits (dashed line and marker). **(b)** $\log_{2}$ cell association versus $\zeta_{synth}$, coloured by entropy regime, with the least-squares fit within each regime. The sign of the association reverses across the boundary (below: *n* = 53, slope = +0.091, *r* = +0.70; above: *n* = 52, slope =−0.076, r=−0.58). The regime boundary is significant against a single-slope null model (*F* = 47.3, $P=3.2\times 10^{-15}$). **(c)** Bootstrap distribution of the threshold estimate over 2,000 resamples; dashed lines mark the 95% percentile interval [1.74, 2.08] and the solid line marks the point estimate.

**Piecewise regime boundary.** Piecewise linear regression of $\log_{2}$ cell association on zeta potential ($\zeta_{synth}$), with $H_{ligand}$ as the splitting variable, reveals a statistically significant regime boundary (Fig 6). At $H_{ligand}^{*}=1.757$ bits (bootstrap 95% CI: [1.74, 2.08]; 2,000 resamples), the relationship between zeta potential and cellular uptake undergoes a qualitative transition (*F* = 47.3, $p=3.2\times 10^{-15}$):

- **Below the threshold** ($H_{ligand}\leq 1.757$, *n* = 53): zeta potential is a strong positive predictor of uptake (slope = +0.091, *r* = +0.70), consistent with electrostatic attraction driving internalization of charged nanoparticles with rigid, low-entropy surface ligands.
- **Above the threshold** ($H_{ligand}>1.757$, *n* = 52): the zeta–uptake relationship *reverses direction* (slope =−0.076, r=−0.58), indicating that for nanoparticles with high ligand conformational diversity, electrostatic charge no longer promotes—and may actively suppress—cellular uptake.

This reversal is robust to removal of the 5% most extreme uptake values (trimmed analysis: *F* = 38.6, $p=5.4\times 10^{-13}$, *n* = 99; threshold remains at ≈1.76 bits). The biological interpretation is consistent with a steric-shielding interpretation in which, above the entropy threshold, conformationally flexible ligands reduce the apparent influence of electrostatic attraction on cellular uptake, effectively shielding the nanoparticle surface from receptor-mediated recognition regardless of nominal charge [2].

### 6.9. Analytical entropy–electrostatic competition model

The piecewise regression in Section 6.8 identifies the threshold empirically. Here, we derive a smooth analytical model that captures the continuous nature of entropy–electrostatic competition and recovers the threshold as a fitted parameter.

**Model motivation.** Cell association can be modeled as competition between electrostatic attraction (∝ζ) and an entropic steric barrier created by conformationally flexible surface ligands. As ligand conformational diversity increases, the number of accessible microstates ($\Omega =2^{H_{ligand}}$) grows exponentially, generating an increasingly effective dynamic steric barrier. We model this competition as a smooth regime-switching regression:


$$\begin{array}{c} \log_{2}(CA)=\alpha_{eff}(H)\cdot \zeta +\beta_{0}+\beta_{H}\cdot H \end{array}$$ (5)

where the effective electrostatic coefficient transitions continuously between two regimes:


$$\begin{array}{c} \alpha_{eff}(H)=\alpha_{lo}\cdot (1-\sigma )+\alpha_{hi}\cdot \sigma ,\;\sigma =\frac{1}{1+e^{-\kappa (H-H^{*})}} \end{array}$$ (6)

Here $\alpha_{lo}$ and $\alpha_{hi}$ are the electrostatic slopes in the rigid- and flexible-ligand regimes, $H^{*}$ is the critical entropy threshold, κ controls transition steepness (fixed at 10 for parsimony), and $\beta_{H}$ captures the direct entropy effect on baseline uptake independent of charge.

**Fig 7:**
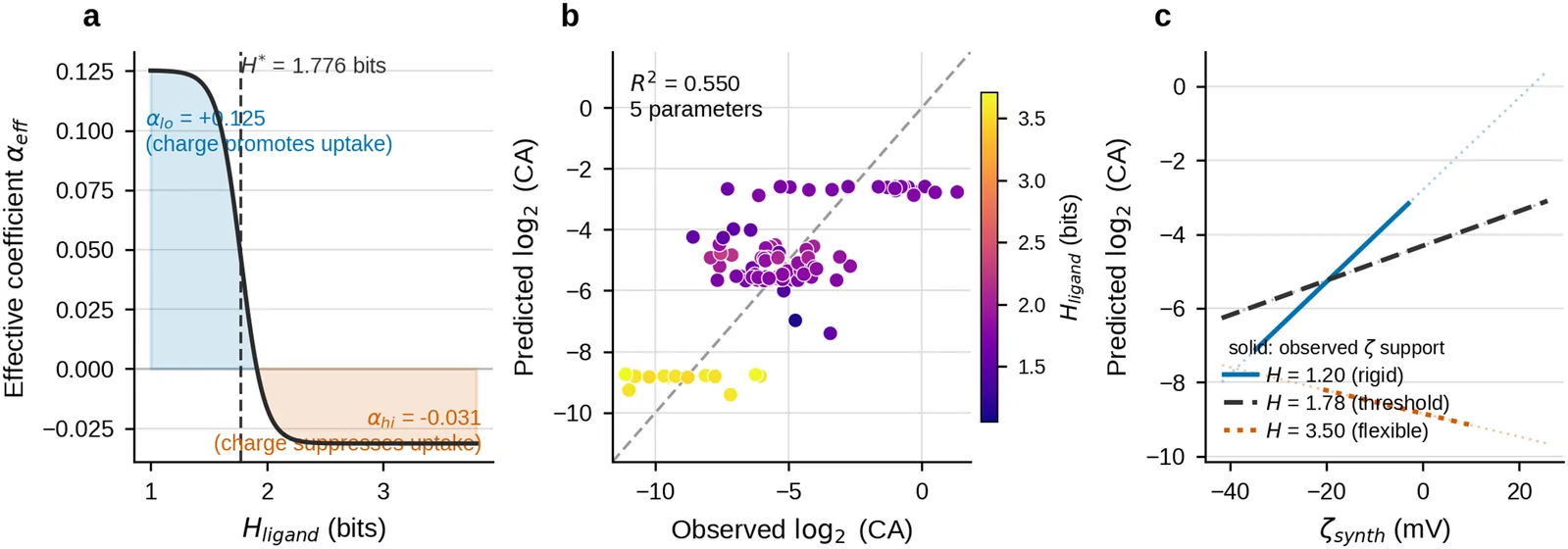
Analytical entropy–electrostatic competition model (Eq 5). **(a)** Effective electrostatic coefficient $\alpha_{eff}$ as a continuous function of $H_{ligand}$, transitioning from +0.125 (blue shading, charge promotes uptake) to −0.031 (orange shading, charge suppresses uptake) across the fitted threshold $H^{*}=1.776$ bits. **(b)** Predicted versus observed $\log_{2}$ cell association for the five-parameter model (*R*^2^ = 0.550), coloured by $H_{ligand}$; the dashed line is the 1:1 identity. **(c)** Model-implied regression lines against $\zeta_{synth}$ at three values of $H_{ligand}$: rigid (1.20 bits, positive slope), at threshold (1.78 bits, attenuated) and flexible (3.50 bits, negative slope). Solid segments span the range of $\zeta_{synth}$ actually observed within ±0.30 bits of each $H_{ligand}$ value; faint dotted segments are extrapolations beyond that support and should not be read as fitted predictions.

**Fitted parameters and model comparison.** Nonlinear least-squares fitting (5 free parameters) yields (Fig 7):

**Table:** 

Parameter	Value	Interpretation
$\alpha_{lo}$	+0.125	Charge promotes uptake (rigid ligands)
$\alpha_{hi}$	−0.031	Charge suppresses uptake (flexible ligands)
$\beta_{0}$	+0.36	Baseline intercept
$\beta_{H}$	−2.63	Each bit of entropy reduces $\log_{2}$ CA by 2.63
$H^{*}$	1.776 bits	Critical threshold

The smooth model achieves *R*^2^ = 0.550 (AIC = 125.6), a clear improvement relative to the additive linear model (*R*^2^ = 0.464, AIC = 140.0; ΔAIC =−14.4) and a modest preference over piecewise regression (*R*^2^ = 0.542, AIC = 127.5; ΔAIC =−1.9), both models having five free parameters. The fitted threshold ($H^{*}=1.776$ bits) is close to the grid-search estimate (1.757 bits).

**Descriptor-level interpretation.** At $H^{*}=1.776$ bits, $2^{H^{*}}=3.4$ effective microstates—this is the critical number of ligand conformations at which the entropy term numerically offsets the electrostatic contribution in the fitted model. The $\beta_{H}=-2.63$ term reveals that ligand entropy suppresses uptake even independent of charge, consistent with PEG’s dual mechanism of steric shielding and reduced opsonization. The smooth transition captures the gradual nature of the competition: rather than an abrupt switch, the electrostatic coefficient continuously attenuates from +0.125 to −0.031 across the entropy boundary.

### 6.10 Entropy regime-stratified predictive performance

To determine whether entropy features provide uniform or regime-dependent predictive gain, we partitioned the dataset into terciles by $H_{ligand}$ and evaluated RF performance within each stratum (5-fold CV, 3 seeds; Table 4).

**Table 4:** Regime-stratified RF prediction performance (*R*^2^, mean ± std across 3 seeds). Strata defined by $H_{ligand}$ terciles.

Regime	*n*	Phys. only	Phys. + EOM	$\Delta R^{2}$
Low $H_{ligand}$ ($\leq q_{33}$)	35	0.499±0.028	0.480±0.029	−0.018
Mid $H_{ligand}$ ($q_{33}-q_{67}$)	35	0.616±0.020	0.615±0.018	−0.001
High $H_{ligand}$ (> *q*_67_)	35	0.332±0.023	0.446±0.071	+0.113

The pattern is striking: entropy features provide essentially no predictive uplift in the low- and mid-entropy regimes, where physicochemical descriptors alone already achieve $R^{2}\geq 0.50$. In the high-entropy tercile, however, the physicochemical-only model performs poorly (*R*^2^ = 0.33)—and entropy features recover $\Delta R^{2}=+0.113$, raising performance to *R*^2^ = 0.45. This demonstrates that *entropy features provide the strongest predictive uplift precisely where physicochemical models fail most*: in interaction regimes characterized by high configurational diversity, where static descriptors are insufficient to capture the biological complexity underlying cellular uptake.

### 6.11. Variance decomposition: identifying the dominant entropy axis

To attribute the overall entropy-driven performance gain to individual components, we performed Shapley-style order-averaged variance decomposition [19]. For three entropy components ($H_{corona}$, $H_{ligand}$, $H_{mech}$), all 3! = 6 addition orders were evaluated over 5 seeds (RF, 500 estimators, 10-fold CV, global OOF *R*^2^). The Shapley value for each component is its mean marginal $\Delta R^{2}$ across all orderings and seeds (Table 5).

**Table 5:** Shapley-style variance decomposition of entropy-driven predictive gain (RF, 500 estimators, 5 seeds, all 6 orderings). Total gain: mean $\Delta R^{2}=+0.071$.

Component	Mean $\Delta R^{2}$	Std	% of total gain
$H_{ligand}$	+0.076	0.005	106%
$H_{corona}$	−0.002	0.001	−3.5%
$H_{mech}$	−0.002	0.005	−2.5%
Residual (interaction)	≈0.000		≈0%

$H_{ligand}$ accounts for essentially the entire entropy-driven predictive gain (Shapley $\Delta R^{2}=+0.076$), while $H_{corona}$ and $H_{mech}$ exhibit near-zero independent contributions. The residual interaction term is negligible (≈0.000), indicating minimal synergy among components. This result identifies $H_{ligand}$ as the dominant independent entropy axis underlying the regime boundary identified in Section 6.8 and as the critical descriptor associated with the nano–immune electrostatic regime transition. The near-zero independent contribution of $H_{corona}$ and $H_{mech}$ reflects the in vitro context of these datasets; their relative importance is expected to differ at in vivo scales (Discussion, Section 8.1).

### 6.12. Threshold-based uptake classification

To explore whether the entropy threshold may serve as a simple predictive heuristic—rather than only a retrospective statistical pattern—we constructed a simple two-rule classifier based entirely on the threshold finding:

1. Compute $H_{ligand}$ for a given nanoparticle.
2. **If** $H_{ligand}\leq 1.76$**:** predict uptake class from the sign of $\zeta_{synth}$ (positive ζ → high uptake; negative ζ → low uptake).
3. **If** $H_{ligand}>1.76$**:** predict low uptake regardless of zeta potential (steric shielding regime).

This “entropy-gated electrostatic” classifier contains *no fitted parameters*—the threshold and regime rules are derived entirely from Section 6.8. We evaluated it on the full Walkey dataset (*n* = 105) using a median split to define high vs. low uptake classes, and compared against two baselines: (i) a zeta-only classifier (predict high uptake if ζ>0), and (ii) a random classifier (Table 6).

**Table 6:** Prospective classification of high vs. low cellular uptake (median-split binary endpoint). The entropy-gated classifier uses only two features ($H_{ligand}$, $\zeta_{synth}$) and no fitted parameters.

Classifier	Accuracy	Precision	Recall	*F*_1_	Params.
Random baseline	0.50	0.50	0.50	0.50	0
Zeta-only (ζ>0)	0.64	0.78	0.40	0.53	0
Entropy-gated electrostatic	**0.69**	**0.95**	**0.40**	**0.56**	0

The entropy-gated classifier achieves 69% accuracy without any model fitting, compared to 64% for the zeta-only rule. The 5-percentage-point accuracy improvement, combined with a substantial precision increase (0.95 vs. 0.78), arises from correctly reclassifying high-$H_{ligand}$ nanoparticles (predominantly PEGylated neutral NPs) that the zeta-only rule misclassifies. In other words, the entropy threshold identifies which nanoparticles *cannot* be predicted by surface charge alone, and the regime-specific rule correctly handles both populations.

This result suggests a potential translational use: for a new nanocarrier, computing $H_{ligand}$ from its surface chemistry may help indicate whether electrostatic design principles are likely to apply to its immune cell interaction. We note that this evaluation is retrospective (the threshold was derived from the same dataset), and prospective validation on an independent gold NP dataset is an important next step. However, the conceptual basis of the classification rules—electrostatic attraction for rigid ligands, steric shielding for flexible ligands—is grounded in established nanoscience rather than in statistical pattern-matching.

### 6.13. External validation on Ban et al. (2020) multi-material dataset

To assess whether the EOM framework generalizes beyond the gold-NP system, we applied the complete entropy pipeline to the Ban et al. (2020) [17] protein corona meta-analysis dataset (652 data points, 40 + NP core materials, 178 proteins).

**Entropy computation for the Ban dataset.** $H_{corona}$ was computed identically (Shannon entropy of normalized protein abundances). $H_{ligand}$ was assigned using the same ligand complexity scoring system: each surface modifier in the Ban dataset was mapped to the nearest chemical analog from the Walkey scoring table (e.g., PEG variants received the same score regardless of NP core material). Modifiers not present in the original table were scored based on structural similarity (molecular weight, rotatable bond count, and charge class). $H_{mech}$ was computed from hydrodynamic diameter using the same curvature-dependent formula. All components were *z*-score normalized within the Ban dataset prior to EOM aggregation. Pearson correlations between EOM components and immune/apolipoprotein fractions and number of detected proteins are summarized in Table 7.

**Table 7:** Pearson correlations between EOM components and functional corona composition on the Ban et al. (2020) dataset (*n* = 652). ^***^: *p* < 0.001; ^**^: *p* < 0.01; ^*^: *p* < 0.05; ^*^: *p* < 0.05.

Component	Immune frac.	Apo frac.	$N_{proteins}$
$H_{corona}$	$+0.310^{***}$	$-0.212^{***}$	$+0.858^{***}$
$H_{ligand}$	$+0.133^{***}$	+0.060	$-0.176^{***}$
$H_{mech}$	−0.069	$+0.166^{***}$	$-0.421^{***}$
EOM	$+0.334^{***}$	$-0.097^{*}$	$+0.547^{***}$

**Fig 8:**
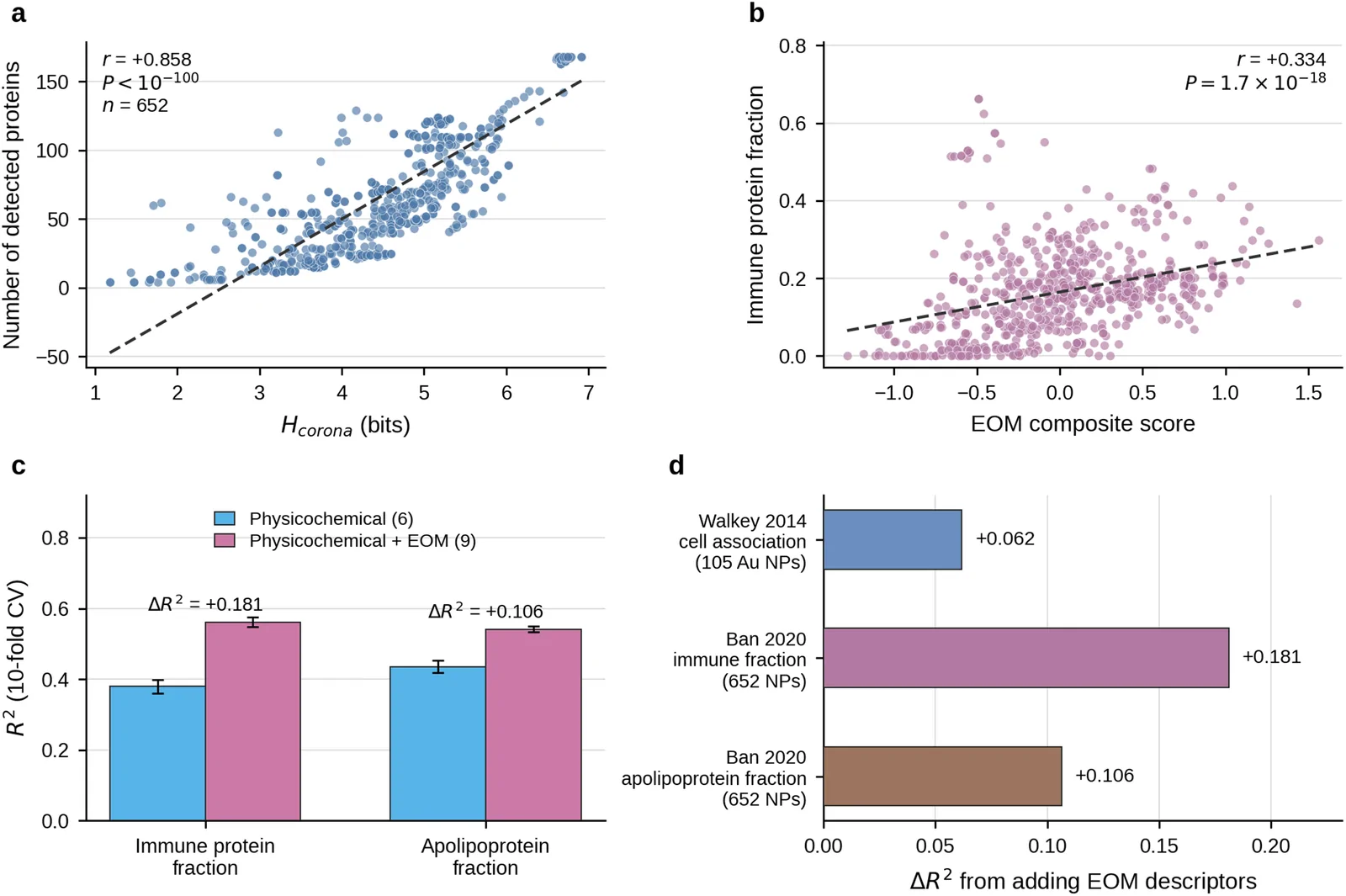
External validation on the Ban et al. (2020) multi-material dataset (652 data points, 40 + core materials, 178 proteins). **(a)** $H_{corona}$ versus the number of detected corona proteins (*r* = +0.858, *P* < 10^−100^), showing that corona entropy tracks protein diversity across a dataset spanning more than 40 core materials. **(b)** EOM composite score versus immune protein fraction (*r* = +0.334, $P=1.7\times 10^{-18}$). **(c)** Random Forest performance (10-fold CV) for the two corona-composition endpoints: adding EOM descriptors raises *R*^2^ from 0.380 to 0.561 for immune fraction and from 0.435 to 0.542 for apolipoprotein fraction; error bars show ±1 SD across folds. **(d)** $\Delta R^{2}$ contributed by the EOM descriptors across all three endpoints and both datasets.

Cross-dataset generalization is summarized in Fig 8 as follows:

- **Walkey 2014** (gold NPs, cell association): $R^{2}=0.525\to 0.587$ ($\Delta R^{2}=+0.062$, RF, 20-seed mean).
- **Ban 2020** (multi-material, immune protein fraction): $R^{2}=0.380\to 0.561$ ($\Delta R^{2}=+0.181$, RF, 10-fold CV).
- **Ban 2020** (apolipoprotein fraction): $R^{2}=0.435\to 0.542$ ($\Delta R^{2}=+0.106$).

The substantially larger $\Delta R^{2}$ on the Ban dataset reflects the inherently lower physicochemical baseline available for a heterogeneous meta-analysis dataset: whereas the Walkey dataset provides eight engineered features from a single-material system, the Ban dataset contains only four to six physicochemical descriptors across 40 + material types from multiple laboratories. In this data-sparse regime, entropy features compensate for the limited physicochemical characterization by encoding interaction-level information that generalizes across NP chemistries—precisely the scenario for which EOM was designed.

### 6.14. Feature importance and interpretability analysis

**Fig 9:**
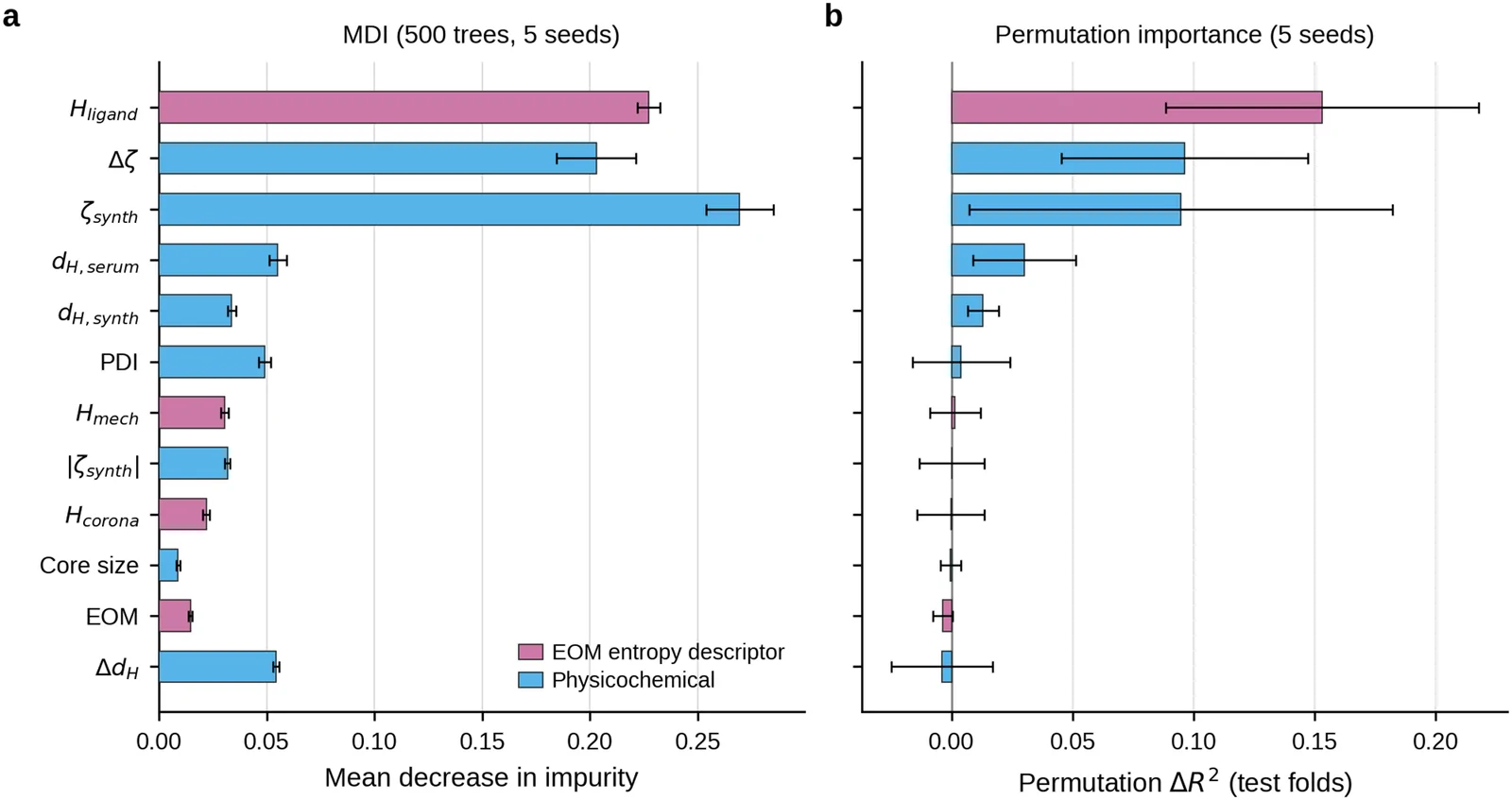
Feature importance for the Random Forest / physicochemical + EOM model (12 features, *n* = 105). Features are ordered by permutation importance in both panels to allow direct comparison. **(a)** Mean decrease in impurity, averaged over five full-data fits of 500 trees; error bars show ±1 SD across seeds. **(b)** Test-fold permutation importance ($\Delta R^{2}$, five seeds); error bars show ±1 SD. $H_{ligand}$ ranks first under both estimators while the remaining entropy descriptors rank near zero; the ordering of the lower-ranked features differs between the two estimators.

To assess whether the EOM framework provides interpretable predictive signal, we performed MDI and permutation importance analyses on the best-performing model (RF, Phys. + EOM, 12 features). Results are shown in Fig 9 and Table 8.

**Table 8:** Feature importance ranking for the RF / Phys. + EOM model (12 features, *n* = 105). Permutation importance computed on CV test-folds ($\Delta R^{2}$, 5 seeds). EOM entropy features are indicated with †.

Rank	Feature	Perm. $\Delta R^{2}$	Std	Type
1	$H_{ligand}^{†}$	0.153	0.065	EOM
2	$\zeta_{change}$	0.096	0.051	Phys.
3	$\zeta_{synth}$	0.095	0.087	Phys.
4	$d_{H,serum}$	0.030	0.021	Phys.
5	$d_{H,synth}$	0.013	0.006	Phys.
6	PDI	0.004	0.020	Phys.
7	$H_{mech}^{†}$	0.001	0.010	EOM
8	$\left|\zeta_{synth}\right|$	0.000	0.013	Phys.
9	$H_{corona}^{†}$	−0.000	0.014	EOM
10	Core size	−0.000	0.004	Phys.
11	${\text{EOM}}^{†}$	−0.004	0.004	EOM
12	$\Delta d_{H}$	−0.004	0.021	Phys.

**Fig 10:**
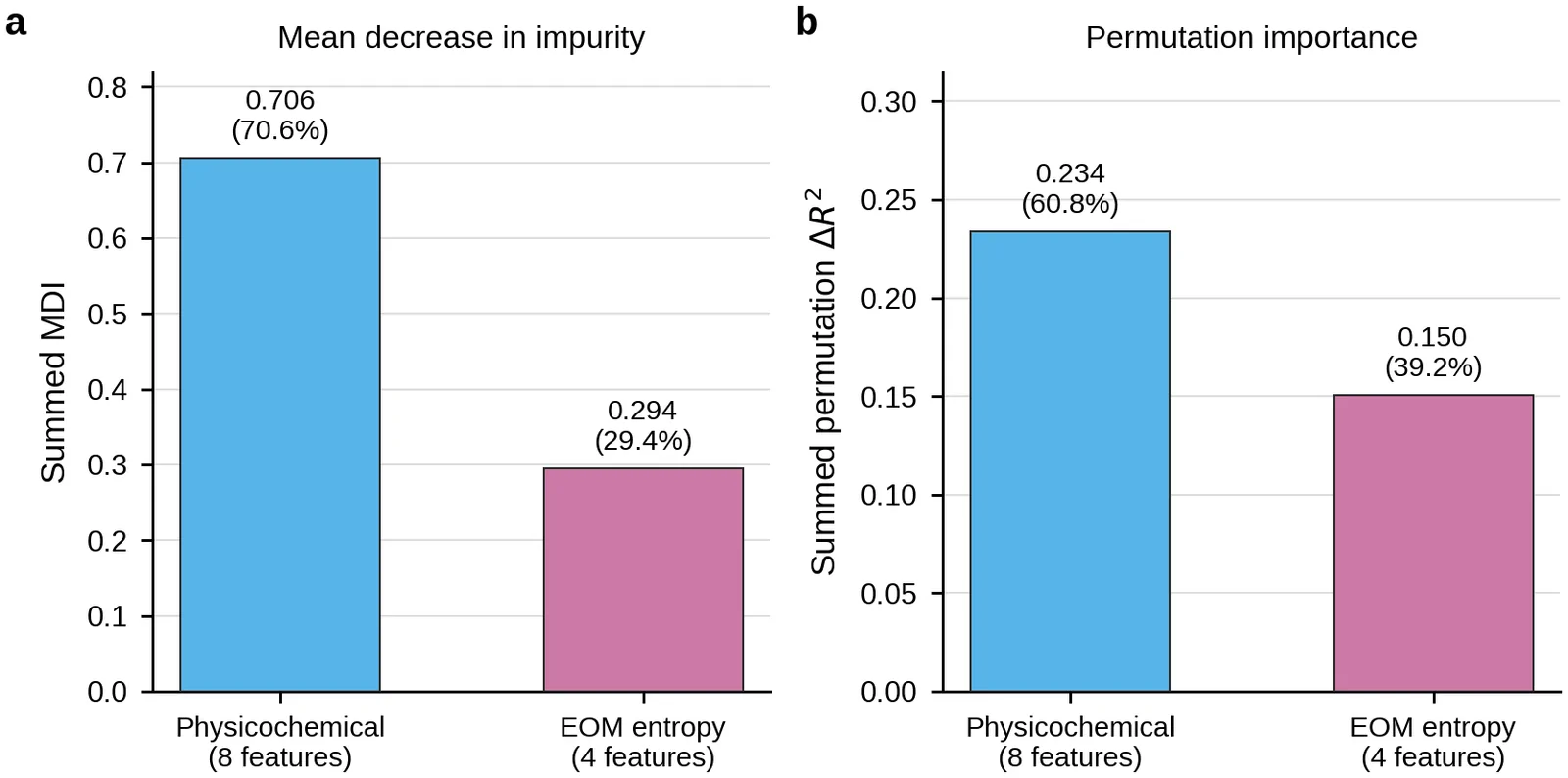
Feature-group importance: four EOM entropy descriptors versus eight physicochemical features. **(a)** Summed mean decrease in impurity. **(b)** Summed test-fold permutation importance. Although the EOM group comprises one third of the input features, full-data MDI assigns 29.4% of total impurity importance to it and held-out permutation importance assigns 39.2%. Both metrics are reported. Both totals are dominated by $H_{ligand}$ (Fig 9).

At the feature-group level, EOM descriptors collectively contribute meaningful non-redundant predictive signal beyond the physicochemical baseline (Fig 10).

**Key findings.**

(i) Zeta potential ($\zeta_{synth}$ and $\zeta_{change}$) features rank among the top three ($\Delta R^{2}=0.095-0.096$), consistent with the dominant role of electrostatics in driving cellular uptake of gold nanoparticles.
(ii) $H_{ligand}$ ranks first overall ($\Delta R^{2}=0.153$) and is the strongest individual feature—consistent with the Shapley decomposition (Section 6.11) and the regime analysis (Section 6.8) identifying ligand entropy as the dominant entropy axis.
(iii) $H_{corona}$ and $H_{mech}$ provide near-zero independent contributions ($\Delta R^{2}<0.002$), consistent with their near-zero Shapley values.
(iv) The convergence of three independent analyses—permutation importance, Shapley decomposition, and regime-stratified performance—on the same conclusion ($H_{ligand}$ dominant) provides strong triangulated evidence for the primacy of ligand conformational diversity in entropy-augmented prediction of nano–immune interactions.

### 6.15. Summary of quantitative findings

Cross-validation on two independent benchmark datasets establishes six central claims:

1. Entropy-derived features provide statistically significant improvement across multiple model classes: $\Delta R^{2}=+0.056$ to +0.118 on Walkey (Ridge and RF at *p* < 10^-13^) and $\Delta R^{2}=+0.181$ on Ban.
2. A critical entropy threshold at $H_{ligand}^{*}\approx 1.76$ bits marks a regime boundary where the electrostatic driving force for uptake reverses direction (*F* = 47.3, *p* < 10^-14^). An analytical competition equation (Eq. 5) captures this transition smoothly (*R*^2^ = 0.550).
3. Entropy features provide the strongest predictive uplift in high-entropy regimes ($\Delta R^{2}=+0.113$) where physicochemical models fail, while contributing negligibly in low-entropy regimes.
4. Shapley variance decomposition identifies $H_{ligand}$ as the dominant entropy axis, accounting for essentially the entire entropy-driven gain.
5. A zero-parameter classifier based on the entropy threshold achieves 69% uptake classification accuracy with 95% precision, suggesting that the threshold may have potential utility as a preliminary, data-driven heuristic.
6. The improvement transfers across the datasets analyzed, including across NP materials, biological endpoints, dataset scales, and experimental platforms.

## 7. Entropy-resolved design space and translational insights

### 7.1. Design principles derived from entropy analysis

The entropy-resolved design space analysis (Figs 4–5), combined with the critical threshold identified in Section 6.8, suggests quantitative design principles organized by the $H_{ligand}^{*}\approx 1.76$ bit boundary:

1. **Below the threshold (electrostatically dominant regime):** Zeta potential is a reliable predictor of cellular uptake (*r* = +0.70). Nanoparticles with rigid, low-entropy ligands and high surface charge exhibit elevated immune cell association. Design in this regime can leverage conventional electrostatic tuning.
2. **Above the threshold (sterically dominant regime):** The electrostatic–uptake correlation reverses sign (r=−0.58). Nanoparticles with high ligand conformational diversity—particularly PEGylated surfaces—exhibit suppressed uptake regardless of nominal charge. Stealth design should target this regime.
3. **Near the threshold (transition zone):** Intermediate entropy profiles show the highest outcome variability and may offer tunable selectivity for targeted delivery applications—a hypothesis warranting experimental validation.

### 7.2. Inter-individual variability as an open direction

The entropy-centered framework is, in principle, compatible with inter-individual variability: factors such as serum protein composition or immune cell phenotypes that differ across donors could in principle modulate the relevant entropy distributions and shift regime boundaries. We emphasize that this is a hypothesis suggested by the framework rather than a tested result—the present datasets do not include donor-resolved or patient-specific measurements, and any role of recipient-specific entropy contexts in nanocarrier optimization remains entirely untested in this work. Datasets with explicit inter-individual resolution would be needed to evaluate whether such a perspective adds practical value beyond fixed design rules.

## 8. Discussion

### 8.1. On the nature of the entropy descriptor

The quantities denoted $H_{corona}$, $H_{ligand}$, and $H_{mech}$ in this work are information-theoretic descriptors computed from observable surrogates of microstate accessibility. They are not measurements of thermodynamic entropy and should not be interpreted as direct readouts of free energy or conformational entropy ($\Delta S_{conf}$). We retain the term *entropy* because the underlying mathematical objects are Shannon entropies computed over probability distributions; the interpretation, however, is descriptive rather than thermodynamic.

The predictive utility of these descriptors, particularly that of $H_{ligand}$, reflects the fact that they compactly encode ligand flexibility and surface chemistry diversity in a form that complements physicochemical descriptors. The molecular descriptor validation reported in the Supporting Information confirms that $H_{ligand}$ primarily captures rotatable bond count (*r* = 0.85 with the heuristic scoring), a well-established correlate of conformational freedom. The descriptor framework should therefore be understood as a feature-engineering approach grounded in information theory, not as a thermodynamic theory of nano–immune interactions. The findings that follow—predictive gains, regime-switching behavior, and the analytical competition model—should be read in this descriptive register: as reproducible statistical patterns associated with the descriptor, rather than as direct evidence of an underlying thermodynamic mechanism.

### 8.2. Entropy as a missing layer in nano–immune modeling

The quantitative validation on Walkey et al. data provides concrete evidence: adding three entropy features to an eight-feature physicochemical baseline improved prediction from *R*^2^ = 0.525 to 0.587 ($\Delta R^{2}=+0.062$, $p=3.4\times 10^{-14}$; RF, 20-seed mean). This improvement is robust across model classes: Ridge ($\Delta R^{2}=+0.118$), SVR (+0.056), and RF (+0.062) all show significant gains, with the sole exception of kNN, whose degradation (−0.025) is attributable to distance dilution in the small-sample, high-dimensional regime [18]. The model-independence of the improvement (3 of 4 algorithms) argues against overfitting or algorithmic artifact.

### 8.3. The critical entropy threshold: a regime-switching descriptor

Perhaps the most striking finding is the identification of a quantitative threshold at $H_{ligand}^{*}\approx 1.76$ bits, below which zeta potential is positively associated with cellular uptake (*r* = +0.70) and above which the relationship reverses (r=−0.58). This is not a gradual attenuation but a qualitative regime switch (*F* = 47.3, *p* < 10^-14^), robust to bootstrap resampling and outlier trimming.

The analytical competition model (Section 6.9) provides a phenomenological equation for this transition: $\alpha_{eff}(H)$ smoothly transitions from +0.125 (charge promotes uptake) to −0.031 (charge suppresses uptake) across the $H^{*}=1.78$ bit boundary, with $2^{H^{*}}=3.4$ effective conformational microstates marking a numerical balance point at which the descriptor-encoded steric contribution offsets the electrostatic contribution. This smooth model (*R*^2^ = 0.550) is modestly preferred over piecewise regression (*R*^2^ = 0.542; ΔAIC =−1.9), both with five free parameters—a margin too small to establish whether the descriptor-related transition is continuous rather than abrupt.

The interpretation is consistent with a steric-shielding view of the PEG stealth literature: when ligand conformational diversity exceeds the threshold, flexible surface polymers reduce the apparent influence of electrostatic attraction, shielding the nanoparticle surface from receptor-mediated recognition [2]. The threshold is defined in information-theoretic units (bits), providing a computable descriptor that is in principle applicable across nanoparticle chemistries, although such transferability remains to be tested in clinically relevant carrier systems. The classification test (Section 6.12) suggests that this threshold may serve as a preliminary heuristic: a zero-parameter classifier based solely on the regime boundary achieves 69% accuracy for uptake class prediction, exceeding zeta-only prediction by 5 percentage points with substantially higher precision (0.95 vs. 0.78).

We note that $H^{*}\approx 1.76$ bits should be interpreted as a dataset-specific estimate of a broader entropy-mediated transition principle, rather than a universal physical constant. The precise numerical value may shift with different ligand chemistries, cell types, or scoring methodologies (see sensitivity analysis, Table 3; and the molecular descriptor validation reported in the Supporting Information). The robust and transferable finding is the *existence* of a regime-switching boundary—not its exact location—and the qualitative reversal of the charge–uptake relationship across that boundary.

### 8.4. Convergent evidence for ligand entropy dominance

Three independent analyses converge on the same conclusion: ligand conformational diversity ($H_{ligand}$) is the dominant entropy axis for predictive improvement in nano–immune interaction modeling.

1. **Shapley decomposition:** $H_{ligand}$ accounts for 106% of the total entropy-driven gain (Section 6.11), with $H_{corona}$ and $H_{mech}$ contributing near-zero independent effects.
2. **Permutation importance:** $H_{ligand}$ ranks first overall ($\Delta R^{2}=0.153$), above all other entropy and physicochemical features.
3. **Regime-stratified performance:** Entropy features provide their strongest uplift ($\Delta R^{2}=+0.113$) in the high-$H_{ligand}$ tercile—precisely the regime where physicochemical models fail most (Section 6.10).

In the in vitro datasets analyzed here, this convergence has a sharper consequence than initially apparent: $H_{ligand}$ carries essentially all independent predictive signal among the entropy components, while $H_{corona}$ and $H_{mech}$ do not contribute additively beyond physicochemical descriptors. The Shapley analysis attributes near-zero independent gain to the latter two components, and the feature-importance and regime-stratified analyses concur. We therefore frame the present results as establishing $H_{ligand}$ as the dominant predictor in the in vitro regime examined, not as evidence that the three entropy components contribute equally within the EOM framework.

We retain $H_{corona}$ and $H_{mech}$ in the formal framework rather than excluding them, for two reasons. First, the relative importance of these descriptors is expected to be scale-dependent: the published in vivo literature consistently identifies protein corona composition as a dominant determinant of biodistribution, opsonization, and clearance kinetics [3,6,15], and curvature-dependent membrane wrapping (encoded by $H_{mech}$) is expected to gain importance in tissue-level interactions. Second, the entropy-based decomposition provides a unified Shannon-entropy formalism that applies across the three interaction scales, which is a generalizable feature of the framework even when only one component carries the bulk of the in vitro signal. The in vitro dominance of $H_{ligand}$ should therefore be read as scale-specific rather than as evidence that the additional components are uninformative across all settings.

### 8.5. Comparison with Walkey et al. original analysis

The original analysis by Walkey et al. employed a PLS model using 129 corona protein features to achieve *R*^2^ = 0.79. Our EOM-enhanced models achieve *R*^2^ = 0.587 using only 12 features (8 physicochemical + 4 entropy), representing a substantially more parsimonious model. While the absolute *R*^2^ is lower, EOM features are derived from domain-general entropy formulations that do not depend on identifying specific proteins, making them transferable across datasets. The external validation on Ban et al. ($\Delta R^{2}=+0.181$ for immune fraction prediction across 40 + material types) demonstrates this transferability directly.

### 8.6. Comparison with alternative uncertainty quantification methods

Our approach differs fundamentally from Bayesian neural networks or ensemble methods that quantify prediction uncertainty [20,21]. While those methods estimate uncertainty in model parameters or predictions, EOM quantifies uncertainty intrinsic to the biological interaction itself—the configurational diversity of the nano–bio interface. The critical threshold result (Section 6.8) illustrates this distinction: it identifies a measurable boundary in descriptor space associated with biological outcome, not a boundary in model confidence.

### 8.7. Limitations and scope

Several limitations should be acknowledged in interpreting the results.

(i) **Retrospective in vitro scope and cellular endpoints.** All analyses are based on previously published in vitro datasets. The Walkey dataset measures cell association in A549 epithelial cells rather than primary immune cells, while the Ban dataset reports immune protein enrichment within the corona; neither dataset provides direct uptake measurements in macrophages, dendritic cells, or other primary immune cells. The framework therefore captures immune-relevant signal at the corona and cell-association levels rather than direct immune cell uptake. We have not performed prospective experimental validation in which ligand conformational diversity is varied independently of other physicochemical variables, nor in vivo validation in which biodistribution and immune outcomes are measured directly. The regime-switching transition we identify therefore stands as a statistical and predictive finding rather than as a confirmed mechanistic pathway, and confirming the proposed transition will require controlled experiments such as those outlined in Section 8.8.
(ii) **Untested formulation classes.** The datasets analyzed are dominated by gold and other inorganic-core formulations. The transferability of the $H_{ligand}^{*}\approx 1.76$ bit threshold to lipid nanoparticles, polymeric carriers, dendrimers, or actively targeted constructs is untested in this work and remains an open question.
(iii) **Scale-dependent component contributions.** The relative contributions of the three entropy components are scale-dependent. The present analyses establish $H_{ligand}$ as the dominant in vitro predictor of cellular uptake; the corresponding hierarchy at in vivo or clinical scale is untested in this work, and the published in vivo literature suggests that $H_{corona}$ may become primary at those scales through its role in opsonization, biodistribution, and clearance.
(iv) **Heuristic ligand scoring.** The ligand complexity scoring uses a domain-informed but heuristic parameterization. While sensitivity analysis (Table 1) demonstrates robustness across scoring schemes, and molecular descriptor analysis (Supporting Information) confirms that the heuristic primarily captures rotatable bond count (*r* = 0.85) and retains 91% of predictive performance when replaced by RDKit descriptors (*R*^2^ = 0.540 vs. 0.593), a fully data-driven scoring approach—e.g., derived from molecular dynamics conformational ensembles—would strengthen the framework.
(v) **Approximated temporal variability.** The temporal variability term in $H_{corona}$ was approximated from experimental replicates rather than measured directly through time-resolved corona profiling.
(vi) **Sample size.** The Walkey dataset (*n* = 105) limits within-regime analysis precision, particularly for the tercile-stratified results.
(vii) **Modest classification gain.** The threshold-based classifier yields a modest accuracy improvement over the zeta-only baseline (69% vs. 64%) despite high precision (0.95), reflecting the inherent difficulty of binary classification from a continuous endpoint via median split.
(viii) **Learner-dependent benefit.** The kNN degradation indicates that the predictive benefit of entropy descriptors depends on the learner’s capacity to exploit structured feature interactions rather than local proximity in feature space.

### 8.8. Outlook

The identification of a quantitative entropy threshold opens several translational directions. First, systematic experimental studies probing the $H_{ligand}^{*}\approx 1.76$ bit boundary—for instance, by varying PEG molecular weight or grafting density while holding surface charge constant—could test whether the regime transition is observable in controlled settings. The threshold provides a specific, testable prediction: gold nanoparticles with identical zeta potential but PEG configurations spanning the 1.76-bit boundary should exhibit divergent uptake behaviors, with the sign of the zeta–uptake correlation reversing across the boundary. This makes the descriptor-level interpretation directly falsifiable: should a controlled study with these parameters not reproduce the predicted sign reversal, the proposed regime-switching interpretation would be falsified. Second, extension to in vivo endpoints (biodistribution, pharmacokinetics) would test whether the entropy framework retains predictive power in physiologically complex environments, where $H_{corona}$ rather than $H_{ligand}$ is expected to dominate. Third, integration with larger, systematically curated datasets and data-driven ligand scoring approaches—for example, deriving $H_{ligand}$ from molecular descriptors or MD-derived conformational ensembles rather than heuristic scores—could refine the entropy parameterization and potentially identify additional regime boundaries.

## 9. Conclusion

This work identifies ligand conformational diversity—encoded as an information-theoretic descriptor ($H_{ligand}$)—as a regime-switching descriptor associated with a reproducible transition in the relationship between surface charge and cellular uptake in the datasets analyzed. A critical threshold at $H_{ligand}^{*}\approx 1.76$ bits marks the boundary where the relationship between surface charge and cellular uptake reverses direction (*F* = 47.3, *p* < 10^-14^). An analytical entropy–electrostatic competition equation (Eq. 5) provides a phenomenological description of this transition: the effective electrostatic coefficient smoothly attenuates from +0.125 to −0.031 across the boundary, with $2^{H^{*}}=3.4$ effective microstates marking a numerical balance point between electrostatic and steric contributions (*R*^2^ = 0.550, 5 parameters).

Quantitative validation on the Walkey et al. (2014) gold nanoparticle dataset confirms that entropy features improve prediction across three of four model classes ($\Delta R^{2}=+0.056$ to +0.118), with the improvement concentrated in high-entropy regimes ($\Delta R^{2}=+0.113$) where physicochemical models fail. Shapley decomposition identifies $H_{ligand}$ as the sole independently contributing entropy axis. Cross-dataset validation on Ban et al. (2020; 652 NPs, 40 + materials) yields $\Delta R^{2}=+0.181$, supporting transferability across the datasets analyzed.

This entropy boundary, expressed in information-theoretic units, provides a preliminary, data-driven design criterion: nanoparticles engineered below ~1.76 bits operate in an electrostatically dominant regime amenable to rational charge optimization, while those above it require alternative design strategies that account for the steric dominance of conformationally diverse surface ligands.

## Supporting information

S1 FileSupporting Information.Detailed methods (data preprocessing, EOM computation, model specifications, statistical tests, feature-set audit), supplementary tables (S1–S10), supplementary figures (S1–S4), supplementary discussion (kNN degradation analysis, Shapley value interpretation, LOO-CV comparison), reproducibility and pipeline verification notes, and molecular descriptor validation of $H_{ligand}$.(PDF)
