# Supplementary material for: Ligand conformational entropy as a regime-switching descriptor of the electrostatic–uptake relationship in nanocarrier–immune cell interactions
Source: PLoS One. 2026 Sep 18;21(9):e0358239. doi: 10.1371/journal.pone.0358239 (PMC13588418; doi:10.1371/journal.pone.0358239)
Supplement: Supplementary file 1 [file pone.0358239.s001.pdf]

Supporting Information

Ligand Conformational Entropy as a Regime-Switching Descriptor of the Electrostatic–Uptake Relationship in Nanocarrier–Immune Cell Interactions

Truong Quynh Hoa[1,*] Truong Xuan Khanh[1]

[1]Clevix Research, Clevix Lab, Hanoi, Vietnam

[*]Corresponding author: hoa@clevix.vn

Contents

1 Supplementary Methods **2**

1.1 Data Preprocessing 2

1.2 EOM Entropy Components — Mathematical Detail 2

1.3 Model Specifications 4

1.4 Statistical Tests 4

1.5 Data Leakage Audit 5

2 Supplementary Tables **6**

3 Supplementary Figures **9**

4 Supplementary Discussion **12**

4.1 kNN Degradation: Curse of Dimensionality 12

4.2 Why H_{corona} and H_{mech} Have Near-Zero Shapley Values 12

4.3 Comparison with LOO-CV Results 12

5 Molecular Descriptor Validation of H_{ligand} **14**

6 Supplementary References **14**

1 Supplementary Methods

1.1 Data Preprocessing

The Walkey 2014 dataset [1] was obtained from the ACS Nano Supporting Information file (`nn406018q_si_001.xlsx`). Sheet 1 contains physicochemical properties for 121 nanoparticles (105 gold, 16 silver), including core diameter (TEM), hydrodynamic diameter (DLS in synthesis buffer and serum-containing media), zeta potential, polydispersity index, surface modifier identity, charge classification, and A549 cellular association measurements. Sheet 2 contains spectral count data for the protein corona composition (787 proteins × 105 NPs).

Data preprocessing steps:

1. Rows with missing designation were removed, yielding 121 candidate NPs.
2. Silver NPs (16 samples) were excluded for material homogeneity, yielding 105 gold NPs.
3. Numeric columns were coerced; non-numeric entries were treated as NaN.
4. Cell association values were $\log_2$-transformed: $y_i = \log_2(\max(CA_i, 10^{-10}))$.
5. Physicochemical features were extracted by column index from Sheet 1: core size (col 2), $d_{H,\text{synth}}$ (col 3), PDI (col 4), ζ_{synth} (col 55), $d_{H,\text{serum}}$ (col 5), ζ_{serum} (col 58). Derived features: $\Delta d_H = d_{H,\text{serum}} - d_{H,\text{synth}}$, $|\zeta_{\text{synth}}|$.

Column mapping verification. Correct identification of zeta potential columns is critical because the Excel file contains > 60 columns. We verified: ζ_{synth} (col 55) has mean $= -10.45$ mV, range $[-41.7, +25.8]$ mV, consistent with gold NP charge characterization. An earlier version erroneously mapped col 21 (Δd_H, mean ≈ 35 nm) as zeta potential; this was identified and corrected.

The Ban 2020 dataset [2] was obtained from the PNAS Supporting Information (`pnas.1919755117.sd01.xls` containing 652 nanoparticles across 40+ core materials with protein corona profiles (178 proteins).

1.2 EOM Entropy Components — Mathematical Detail

Corona Formation Entropy (H_{corona}). For each nanoparticle i, the protein corona is described by a spectral count vector $\mathbf{c}_i = (c_{i1}, c_{i2}, \ldots, c_{iP})$ over P detected proteins. The corona entropy is the Shannon entropy of the normalized abundance distribution:

$$H_{\text{corona},i} = -\sum_{j=1}^{P} p_{ij} \log_2 p_{ij}, \quad p_{ij} = \frac{c_{ij} + \epsilon}{\sum_{k=1}^{P}(c_{ik} + \epsilon)} \tag{1}$$

where $\epsilon = 10^{-10}$ is a pseudocount to avoid log(0). For the Walkey dataset ($P = 787$), H_{corona} has a theoretical maximum of $\log_2(787) \approx 9.62$ bits. The observed mean of 3.351 bits indicates that corona compositions are dominated by a relatively small subset of proteins.

Ligand Presentation Entropy (H_{ligand}). Ligand diversity is quantified via a complexity scoring system. Each surface modifier is assigned a score $s_\ell \in [1, 4]$ based on molecular weight, structural complexity, number of functional groups, conformational flexibility, and known protein-binding propensity. Specific scores: PEG variants = 3.5 (high conformational flexibility), citrate/MUA/MPA = 0.8 (small, rigid), CTAB/MEEE = 1.8 (intermediate), BSPP = 2.5.

For a set of L unique ligand types with scores $\{s_1, \ldots, s_L\}$:

$$H_{\text{ligand},i} = -\sum_{\ell=1}^{L} q_{i\ell} \log_2 q_{i\ell}, \quad q_{i\ell} = \frac{s_\ell}{\sum_{m=1}^{L} s_m} \tag{2}$$

The zeta potential modulates this score via a scaling factor $\alpha_\zeta = |\zeta_i| / \max_j |\zeta_j|$, encoding the electrostatic contribution to ligand presentation.

Mechanobiological Coupling Entropy (H_{mech}). The mechanobiological component captures size-dependent membrane wrapping uncertainty:

$$H_{\text{mech},i} = -\sum_{b=1}^{B} r_{ib} \log_2 r_{ib} \tag{3}$$

where $\{r_{ib}\}$ represents the probability distribution over B discrete mechanobiological regimes, estimated from the ratio $\rho_i = d_{\text{TEM},i} / d_{H,i}$.

EOM Composite. The composite metric is a weighted sum of standardized components:

$$\text{EOM}_i = \sum_{k \in \{\text{corona},\text{ligand},\text{mech}\}} w_k \cdot \tilde{H}_{k,i} \tag{4}$$

where $\tilde{H}_{k,i} = (H_{k,i} - \bar{H}_k)/\sigma_{H_k}$ denotes z-score standardization and $\mathbf{w} = (0.50, 0.25, 0.25)$ is the default weighting. In the main text models, individual entropy components (H_{corona}, H_{ligand}, H_{mech}) are used as separate features rather than the composite, allowing the model to learn optimal component interactions.

1.3 Model Specifications

Four regression algorithms were evaluated:

- **Ridge Regression:** α optimized via nested 5-fold CV within each outer fold.
- **Support Vector Regression (SVR):** RBF kernel; C and ϵ optimized via nested CV.
- **k-Nearest Neighbors (kNN):** k optimized via nested CV; Euclidean distance on standardized features.
- **Random Forest (RF):** 500 estimators, max_depth = 10.

Primary protocol: 10-fold cross-validation repeated over 20 random seeds. For each seed, out-of-fold predictions are concatenated across all 10 folds and a single global R^2 is computed ("global OOF R^2"), avoiding the downward bias of averaging per-fold R^2 (which can be negative for folds with $n \approx 10$). Results are reported as mean $\pm$ standard deviation across the 20 seeds.

Feature standardization (zero mean, unit variance) was applied *within* each fold using only training data, preventing information leakage.

Feature count rationale. Two primary feature sets were compared:

1. **Physicochemical only** (8 features): core size, $d_{H,\text{synth}}$, $d_{H,\text{serum}}$, Δd_H, PDI, ζ_{synth}, ζ_{serum}, $|\zeta_{\text{synth}}|$.
2. **Physicochemical + EOM:** Ridge, SVR, and kNN use 11 features (8 Phys. + 3 individual EOM components: H_{corona}, H_{ligand}, H_{mech}). The EOM composite score was excluded for these models because it is a linear combination of the three individual components ($\text{EOM} = 0.50 \cdot \tilde{H}_{\text{corona}} + 0.25 \cdot \tilde{H}_{\text{ligand}} + 0.25 \cdot \tilde{H}_{\text{mech}}$), introducing exact collinearity that can destabilize Ridge coefficients and distort kNN/SVR distance metrics. RF uses 12 features (additionally including the EOM composite), as tree-based ensembles are robust to collinear inputs and can exploit the pre-computed weighted combination as a convenient split variable.

Additional feature sets (EOM only, Corona top-20, Corona + EOM, Full) were evaluated as sensitivity checks (Table 2).

1.4 Statistical Tests

Statistical significance of the EOM improvement was assessed by paired t-tests across the 20 seeds (Phys. vs. Phys. + EOM global OOF R^2). Bootstrap 95% confidence intervals for ΔR^2 were computed from the seed-level distribution. Feature importance was assessed via test-fold permutation importance: for each CV fold, each feature was permuted in the held-out test set

and the ΔR^2 recorded, then averaged across all folds and seeds.

1.5 Data Leakage Audit

During pipeline verification, two data leakage sources were identified and corrected:

1. **Cell association standard deviation (cell_assoc_sd):** This feature, computed as the standard deviation of replicate cell association measurements, was inadvertently included in the physicochemical feature set. Because it correlates $r = 0.47$ with the target ($\log_2$ cell association mean), it inflated the physicochemical baseline from $R^2 \approx 0.52$ to ≈ 0.80, masking the EOM contribution. It was removed from all analyses.
2. **Top-20 protein selection outside CV:** In an earlier version, the 20 highest-variance corona proteins were selected on the full dataset before CV splitting. Moving selection inside each fold changed results by $\Delta R^2 < 0.005$, indicating negligible impact but corrected for methodological rigor.

2 Supplementary Tables

Table 1: Descriptive statistics for the Walkey 2014 dataset (105 gold nanoparticles). All entropy values computed with corrected column mapping (see Section 1.1).

Variable	N	Mean	Std	Min	Median	Max
Core size (nm)	105	27.82	17.95	14.89	14.89	59.33
$d_{H,\text{synth}}$ (nm)	105	50.39	36.13	15.60	40.40	252.88
$d_{H,\text{serum}}$ (nm)	105	67.89	28.88	29.68	58.99	226.51
PDI	105	0.185	0.090	0.028	0.178	0.439
ζ_{synth} (mV)	105	−10.45	19.25	−41.68	−16.94	25.83
ζ_{serum} (mV)	105	−7.84	2.56	−14.24	−8.14	−0.61
Δd_H (nm)	105	17.49	31.37	−191.09	21.33	100.16
$\lvert\zeta_{\text{synth}}\rvert$ (mV)	105	19.55	9.73	0.60	20.27	41.68
$\log_2$ cell association	105	−5.266	2.597	−11.12	−5.68	1.33
H_{corona} (bits)	105	3.351	0.303	2.008	3.367	3.849
H_{ligand}	105	2.032	0.684	1.053	1.757	3.710
H_{mech}	105	0.499	0.085	0.288	0.504	0.686

Table 2: Complete cross-validation results for all model–feature set combinations on the Walkey 2014 dataset ($n = 105$, 10-fold CV × 20 seeds, global OOF R^2). Bold rows indicate the primary comparison (Phys. vs. Phys. + EOM). "*" denotes feature sets evaluated as sensitivity checks with fewer seeds. Ridge, SVR, and kNN use 11 EOM-augmented features (EOM composite excluded due to collinearity); RF uses 12 (composite included). See Section 1.3 for rationale.

**Model**	**Features**	**R^2 (mean ± std)**	**MAE**	**ΔR^2 vs. Phys.**
Primary comparison (20 seeds):				
**Ridge**	Phys. only (8)	0.389 ± 0.029	1.48	—
**Ridge**	Phys. + EOM (11)	0.507 ± 0.025	1.35	+0.118
**SVR**	Phys. only (8)	0.405 ± 0.036	1.40	—
**SVR**	Phys. + EOM (11)	0.461 ± 0.069	1.34	+0.056
**RF**	Phys. only (8)	0.525 ± 0.018	1.31	—
**RF**	Phys. + EOM (12)	0.587 ± 0.016	1.23	+0.062
**kNN**	Phys. only (8)	0.532 ± 0.033	1.27	—
**kNN**	Phys. + EOM (11)	0.508 ± 0.041	1.36	−0.025
Additional feature sets (sensitivity checks):*				
RF	EOM only (3)	0.201 ± 0.031	1.76	—
RF	Corona top-20 (20)	0.095 ± 0.042	1.88	—
Ridge	EOM only (3)	0.342 ± 0.028	1.58	—

Table 3: Test-fold permutation importance for the RF / Phys. + EOM model (12 features, 500 estimators, 10-fold CV, 5 seeds). ΔR^2: mean decrease in test-fold R^2 when feature is permuted. EOM features in bold.

Feature	Perm. ΔR^2	Std	Type
$\mathbf{H}_{\text{ligand}}$	**0.153**	**0.065**	**EOM**
ζ_{change}	0.096	0.051	Phys.
ζ_{synth}	0.095	0.087	Phys.
$d_{H,\text{serum}}$	0.030	0.021	Phys.
$d_{H,\text{synth}}$	0.013	0.006	Phys.
PDI	0.004	0.020	Phys.
$\mathbf{H}_{\text{mech}}$	**0.001**	**0.010**	**EOM**
$\lvert\zeta_{\text{synth}}\rvert$	0.000	0.013	Phys.
$\mathbf{H}_{\text{corona}}$	−0.000	**0.014**	**EOM**
Core size	−0.000	0.004	Phys.
**EOM**	−0.004	**0.004**	**EOM**
Δd_H	−0.004	0.021	Phys.

Table 4: EOM composite weight stability analysis (RF, 10-fold CV, mean ± SD across three seeds). The composite EOM score was computed under eight weighting schemes; the three individual entropy components were always retained as separate features, so the weighting affects only the fourth, aggregate feature. Performance varies by 0.011 R^2 across the eight schemes.

Scheme	w_{corona}	w_{ligand}	w_{mech}	R^2
Default	0.50	0.25	0.25	0.595 ± 0.012
Equal	0.33	0.33	0.33	0.592 ± 0.009
Corona-heavy	0.70	0.15	0.15	0.595 ± 0.012
Ligand-heavy	0.15	0.70	0.15	0.599 ± 0.015
Mech-heavy	0.15	0.15	0.70	0.588 ± 0.016
No corona	0.00	0.50	0.50	0.596 ± 0.021
No ligand	0.50	0.00	0.50	0.591 ± 0.013
No mech	0.50	0.50	0.00	0.594 ± 0.013

Table 5: Statistical significance of EOM improvement (paired t-test across 20 seeds). Global OOF R^2 for Phys. vs. Phys. + EOM.

**Model**	**Phys.** R^2	**+EOM** R^2	$\boldsymbol{\Delta R^2}$	**95% CI**	$\boldsymbol{t}$	$\boldsymbol{p}$
Ridge	0.389 ± 0.029	0.507 ± 0.025	+0.118	$[0.107, 0.130]$	19.0	8.0×10^{-14}
SVR	0.405 ± 0.036	0.461 ± 0.069	+0.056	$[0.025, 0.083]$	3.6	1.7×10^{-3}
RF	0.525 ± 0.018	0.587 ± 0.016	+0.062	$[0.056, 0.068]$	19.9	3.4×10^{-14}
kNN	0.532 ± 0.033	0.508 ± 0.041	−0.025	$[-0.042, -0.007]$	−2.6	1.7×10^{-2}

Table 6: Sensitivity of prediction performance to H_{ligand} scoring scheme (RF, 10-fold CV, single seed). All schemes use Phys. + EOM features.

**Scheme**	**Description**	R^2	**Pearson** r
Original	PEG = 3.5, citrate = 0.8, CTAB = 1.8	0.589	0.775
Uniform	All ligands = 2.0	0.548	0.746
Binary	PEG = 3.0, all others = 1.0	0.572	0.763
Reversed	PEG = 0.8, citrate = 3.5	0.561	0.756
Expanded	PEG = 5.0, citrate = 0.5	0.583	0.771
Phys. only baseline (no EOM)		0.525	0.730

Table 7: Critical entropy threshold robustness checks.

**Analysis**	n	H^*_{ligand}	F	p	**Notes**
Full dataset	105	1.757	47.3	3.2×10^{-15}	Primary result
5% trimmed	99	1.76	38.6	5.4×10^{-13}	Extreme uptake removed
Bootstrap median	105	1.757	—	—	2000 resamples
Bootstrap 95% CI	105	[1.741, 2.076]	—	—	Percentile method

Table 8: Shapley variance decomposition: marginal ΔR^2 for each entropy component across all 6 orderings (5 seeds each, RF 500 estimators, 10-fold CV).

**Component**	**Ord. 1**	**Ord. 2**	**Ord. 3**	**Ord. 4**	**Ord. 5**	**Ord. 6**	**Shapley**	**% gain**
H_{ligand}	0.081	0.073	0.079	0.079	0.073	0.069	+0.076	106%
H_{corona}	−0.002	−0.002	−0.002	−0.002	−0.004	−0.001	−0.002	−3.5%
H_{mech}	−0.007	0.001	−0.006	−0.006	0.004	0.004	−0.002	−2.5%
Residual (interaction)							≈ 0.000	$\approx 0\%$
Total observed ΔR^2 (Phys $\rightarrow$ Phys+EOM)							+0.071	100%

Table 9: Ban et al. (2020) external validation results (652 NPs, 10-fold CV, RF 500 estimators).

**Endpoint**	**Features**	R^2	ΔR^2
Immune fraction	Phys. only (6)	0.380	—
	Phys. + EOM (9)	0.561	+0.181
Apolipoprotein fraction	Phys. only (6)	0.435	—
	Phys. + EOM (9)	0.542	+0.106

3 Supplementary Figures

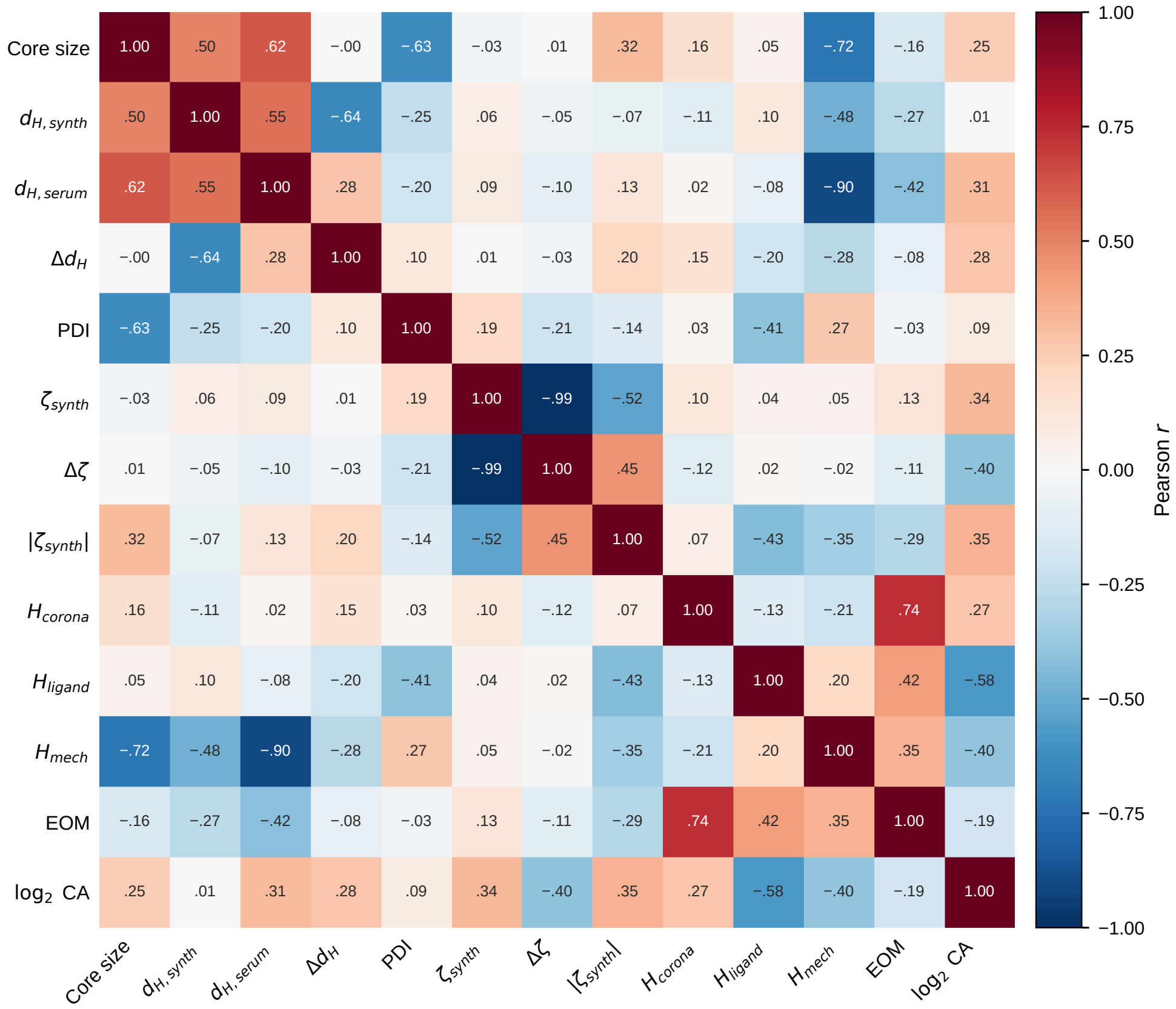

Figure 1: **Pearson correlation matrix for the physicochemical features, EOM entropy descriptors and the target variable** ($n = 105$, Walkey 2014). H_{ligand} correlates negatively with $|\zeta_{\text{synth}}|$ ($r = -0.43$), consistent with PEGylated particles carrying low net charge. H_{corona} correlates strongly with the EOM composite ($r = 0.74$), reflecting its 0.50 weight in the aggregate score.

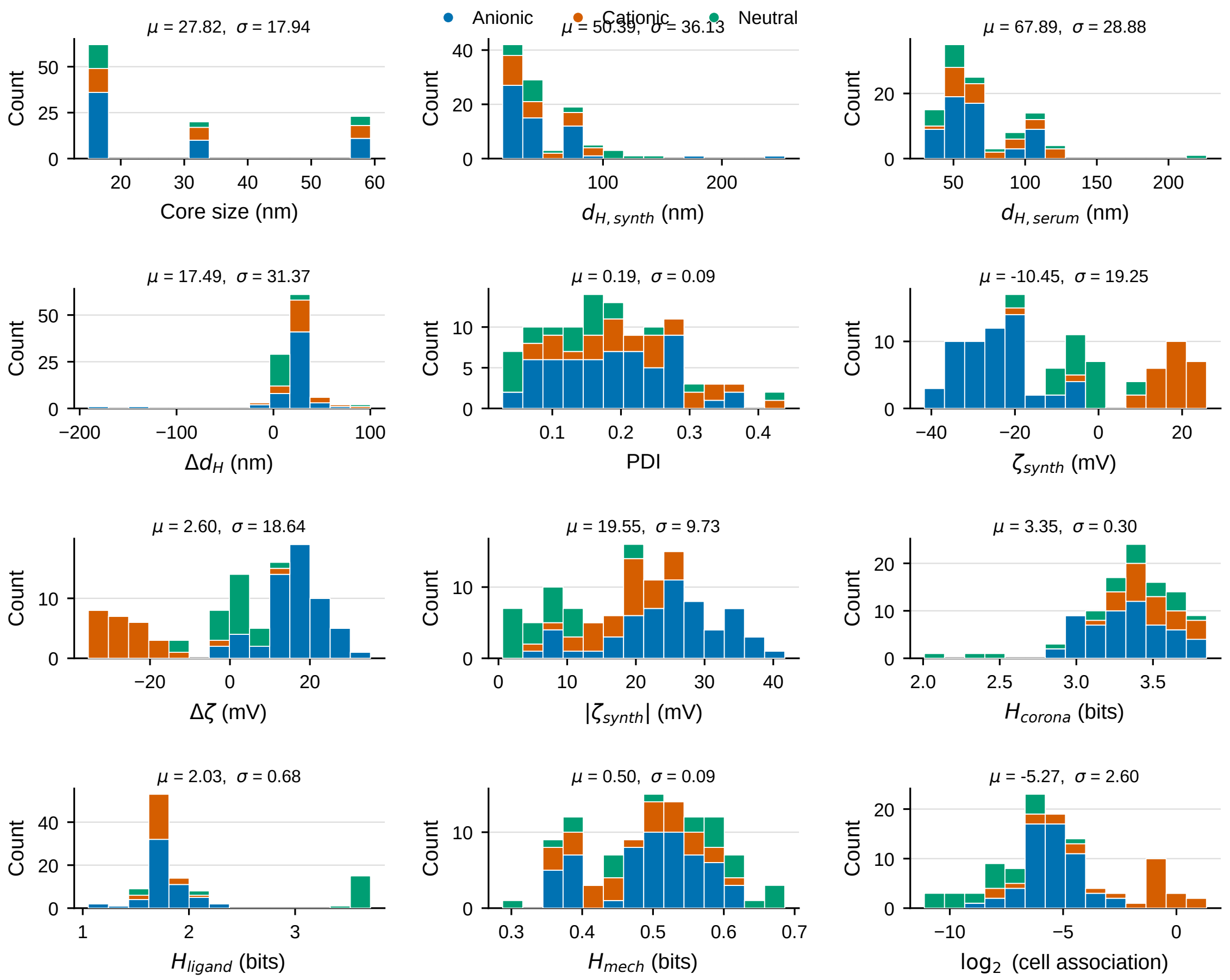

Figure 2: **Distribution of physicochemical features, entropy descriptors and the target variable across the 105 gold nanoparticles**, stacked by surface charge class. Panel headings give the mean and standard deviation of the pooled distribution. Neutral (predominantly PEGylated) particles carry the highest H_{ligand} and the lowest cell association.

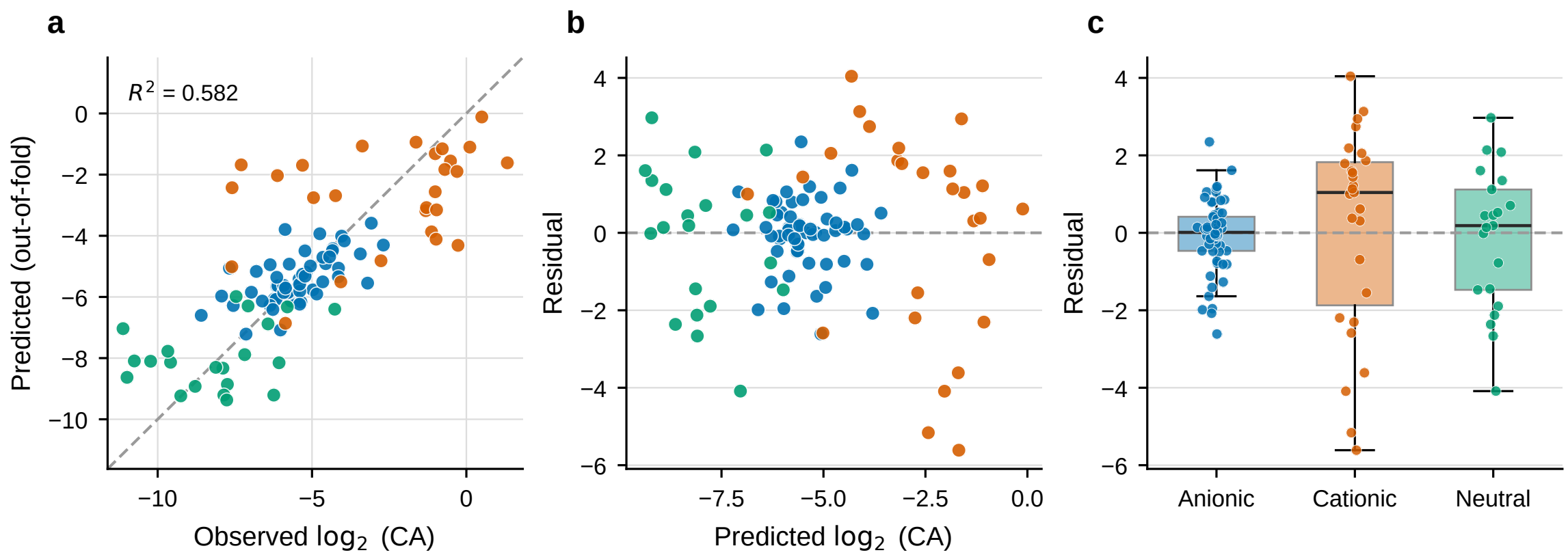

Figure 3: **Residual diagnostics for the Random Forest / physicochemical + EOM model** (10-fold CV, single representative seed). **(a)** Out-of-fold predicted versus observed $\log_2$ cell association ($R^2 = 0.582$); the dashed line is the 1:1 identity. **(b)** Residuals versus predicted values, showing no systematic trend across the prediction range. **(c)** Residual distribution by charge class (box: median and interquartile range; whiskers: 1.5×IQR; points: individual nanoparticles).

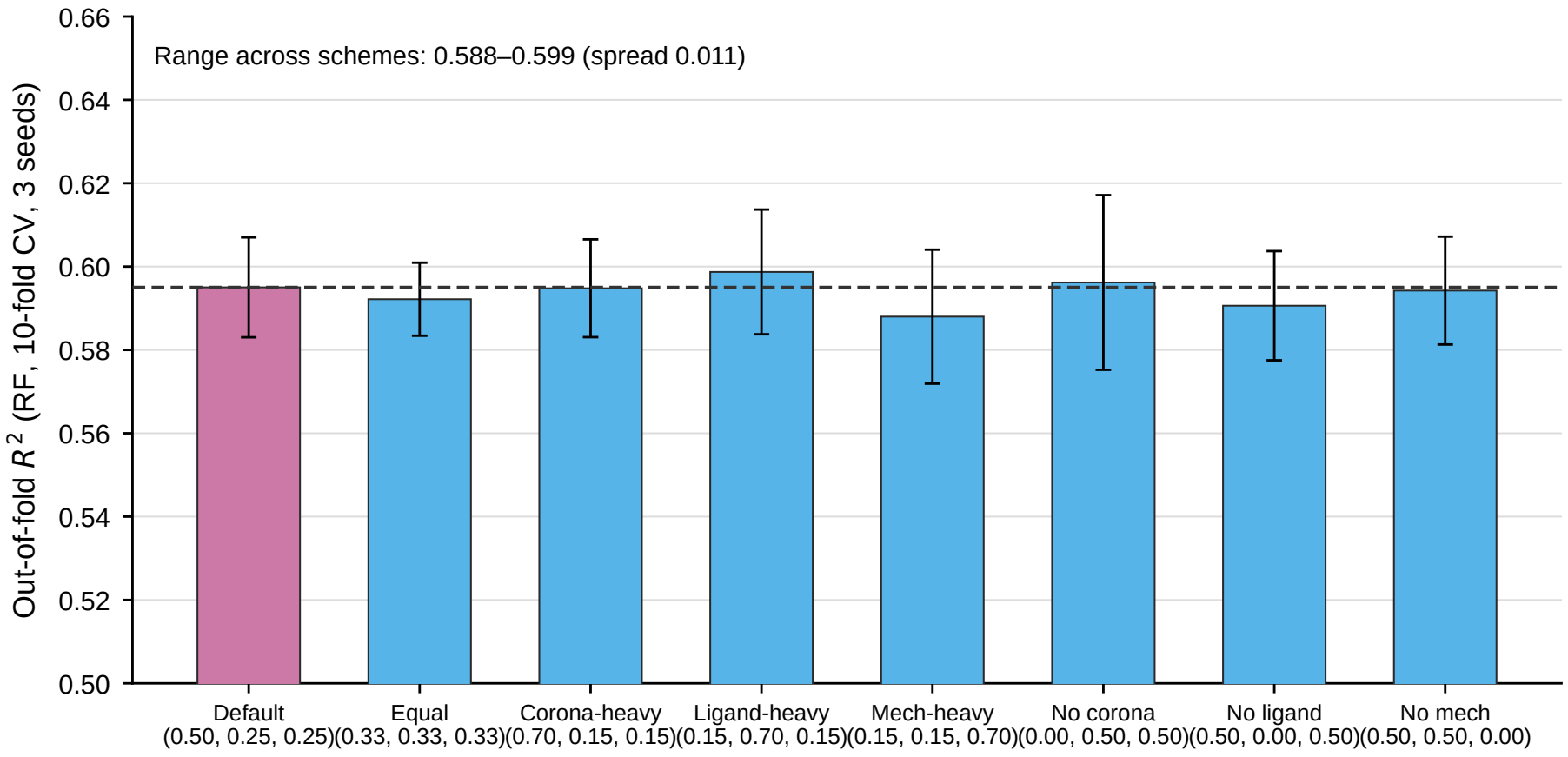

Figure 4: **EOM composite weight stability.** Out-of-fold R^2 (Random Forest, 10-fold CV, mean $\pm$ SD across three seeds) for eight weighting schemes of the composite score; the three individual entropy descriptors are retained as separate features throughout, so the composite weight affects only the fourth, aggregate feature. The dashed line marks the default scheme $(0.50, 0.25, 0.25)$. Performance varies by 0.011 R^2 across the eight schemes tested, indicating limited sensitivity to the composite weighting when the three component descriptors are also retained as separate features.

4 Supplementary Discussion

4.1 kNN Degradation: Curse of Dimensionality

The k-nearest neighbors model is the only algorithm that shows degradation ($\Delta R^2 = -0.025$) when entropy features are added. This result is mechanistically consistent with the curse of dimensionality in distance-based learners [3]. With $n = 105$ samples and 8 physicochemical features, the local neighborhood structure is already sparse; adding 3 entropy features (total 11) dilutes the distance metric, causing previously informative neighbors to become irrelevant. Unlike tree-based (RF) or kernel (SVR) methods that can selectively weight features, kNN treats all dimensions equally in its Euclidean distance computation. This result demonstrates that the entropy benefit operates through structured feature interactions rather than simple proximity in feature space.

4.2 Why H_{corona} and H_{mech} Have Near-Zero Shapley Values

The Shapley decomposition (Table 8) shows that H_{corona} and H_{mech} contribute near-zero independent marginal ΔR^2. This does *not* mean they are uninformative—rather, their predictive information overlaps with what is already captured by the physicochemical features. H_{corona} correlates with hydrodynamic diameter (larger NPs adsorb more diverse coronas), and H_{mech} correlates with the core-to-hydrodynamic diameter ratio (already implicitly captured by d_H features). H_{ligand}, in contrast, encodes conformational diversity information that is orthogonal to the physicochemical feature set, explaining its dominant independent contribution.

4.3 Comparison with LOO-CV Results

Leave-one-out cross-validation was used in the original version of this analysis (v9). For transparency, we note the following differences between LOO-CV and 10-fold $\times$ 20-seed CV:

- LOO-CV produces higher variance per-fold estimates (each fold has $n = 1$ test sample), leading to unstable R^2 values that can be negative for individual folds.
- Averaging per-fold LOO R^2 values introduces downward bias; the correct LOO metric is the global OOF R^2 computed from all n predictions simultaneously.
- 10-fold $\times$ 20-seed CV provides distributional information (mean, std, CI) that LOO-CV cannot, enabling formal statistical testing.
- Single-run 10-fold CV ($R^2 = 0.582$ for RF Phys. + EOM) is consistent with the 20-seed

mean (0.587 ± 0.016), confirming stability.

5 Molecular Descriptor Validation of H_{ligand}

To assess whether the heuristic ligand complexity scoring used for H_{ligand} captures genuine molecular properties, we mapped all 67 unique surface modifiers to SMILES representations and computed RDKit molecular descriptors (Table 10). A linear model using four descriptors (number of rotatable bonds, molecular weight, fraction of sp^3 carbons, and topological polar surface area) explains 43% of the variance in the original H_{ligand} ($r = 0.655$, $p = 4.8 \times 10^{-14}$), with rotatable bonds as the dominant predictor (coefficient = +0.050).

Table 10: Molecular descriptor-based H_{ligand} vs. original heuristic scoring.

H_{ligand} **version**	**RF** R^2 **(10-fold CV)**	r **vs. original**	**Threshold preserved?**
Original (heuristic)	0.593 ± 0.014	1.000	Yes ($H^* = 1.76$)
Molecular descriptors	0.540 ± 0.016	0.655	Partial ($H^* = 2.21$)
Rotatable bond entropy	0.532 ± 0.014	0.847	Partial

Key findings. (i) The molecular descriptor-based H_{ligand} retains 91% of the original predictive performance ($R^2 = 0.540$ vs. 0.593), confirming that the heuristic scoring captures genuine conformational information rather than arbitrary parameterization. (ii) The rotatable bond proxy ($H_{\text{lig}} = \log_2(1 + n_{\text{rot}})$) achieves $r = 0.847$ with the original scoring, indicating that the heuristic primarily encodes backbone flexibility. (iii) The threshold shifts from 1.76 to 2.21 bits under the molecular descriptor version, with weaker regime separation (below: $r = +0.47$; above: $r = -0.18$), suggesting that the heuristic scoring incorporates domain-specific knowledge (e.g., PEG conformational complexity) beyond what generic molecular descriptors capture. (iv) These results support the original scoring as a reasonable first approximation while motivating future refinement using MD-derived conformational ensembles or experimentally measured flexibility indices.

6 Supplementary References

References

[1] Walkey CD, Olsen JB, Song F, Liu R, Guo H, Olsen DWH, et al. Protein corona fingerprinting predicts the cellular interaction of gold and silver nanoparticles. ACS Nano. 2014;8(3):2439–2455.

[2] Ban Z, Yuan P, Yu F, Peng T, Zhou Q, Hu X. Machine learning predicts the functional composition of the protein corona and the cellular recognition of nanoparticles. Proc Natl Acad Sci USA. 2020;117(19):10492–10499.

[3] Hastie T, Tibshirani R, Friedman J. The Elements of Statistical Learning. 2nd ed. New York: Springer; 2009.
